# Synthesis of aryloxyacetamides from arylboronic acids and 2-bromoacetonitrile promoted by alkaline solutions of hydrogen peroxide†

**DOI:** 10.1039/d2ra07451f

**Published:** 2023-01-18

**Authors:** Mengping Guo, Yingmin Li, Yongju Wen, Xiuli Shen

**Affiliations:** a Institute of Coordination Catalysis, College of Chemistry and Bio-Engineering, Yichun University Yichun 336000 PR China guomengping65@163.com

## Abstract

A novel and metal catalyst-free synthesis of aryloxyacetamides from the corresponding arylboronic acids and 2-bromoacetonitrile promoted by alkaline solutions of hydrogen peroxide has been developed involving an oxidation–reduction of eco-friendly H_2_O_2_ with simultaneous reaction *ipso*-hydroxylation of arylboronic acid and hydration of the nitrile. This protocol is compatible with sensitive substituents attached to the arylboronic acid and provides desired products in moderate to good yields in pure water.

## Abstract 2

A novel and metal catalyst-free synthesis of aryloxyacetamides from the corresponding arylboronic acids and 2-bromoacetonitrile promoted by alkaline solutions of hydrogen peroxide has been developed.

## Graphical abstract

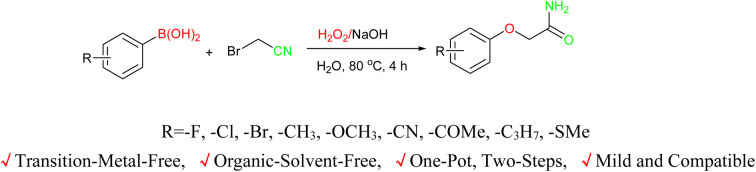

## Introduction

**Scheme 1:**
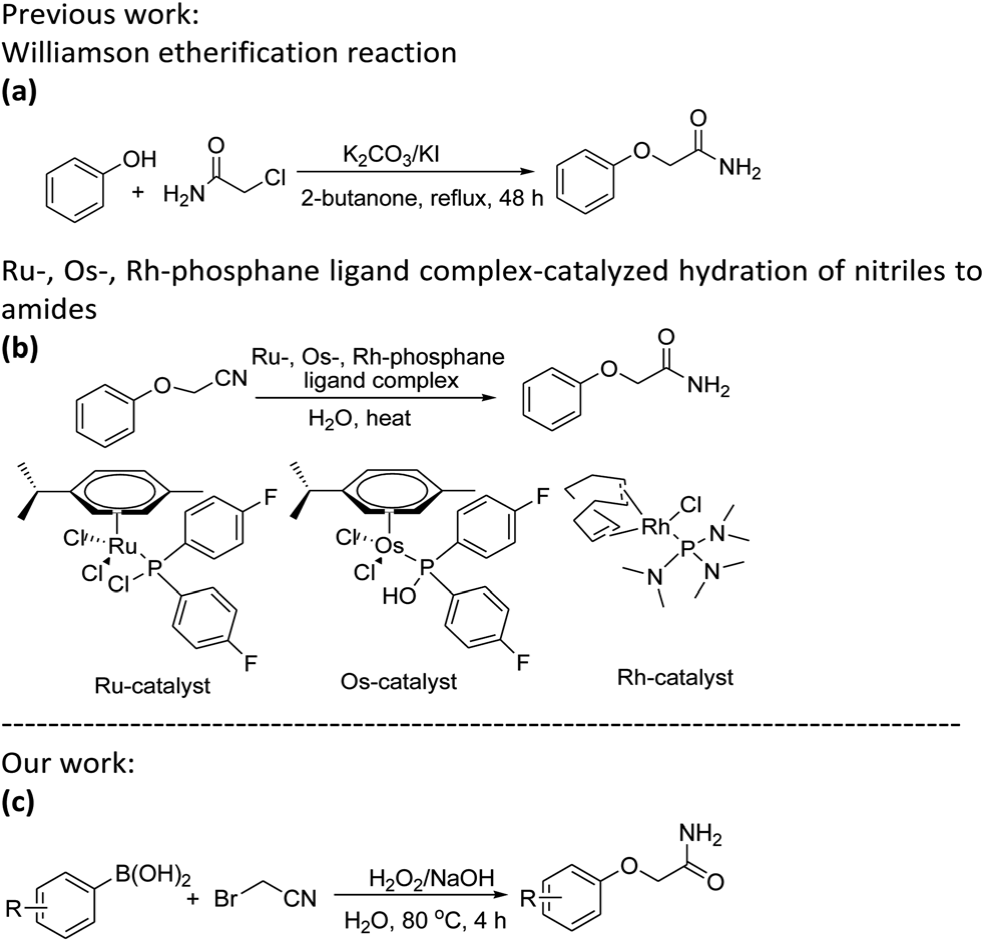
Some synthetic protocols for aryloxyacetamides.

Aryloxyacetamides are significant structural components that have attracted a lot of interest in pharmaceuticals,^1–4^ agrochemicals,^5,6^ and organic synthesis.^7–9^ A minimum of one amide pharmacophore is present in about 25% of all medications on the market today.^10^ As a result, it is now crucial to create concise, inexpensive and environmentally friendly processes for producing aryloxyacetamides. By Williamson etherifying phenol and chloroacetamide in a polar organic solvent, aryloxyacetamides are traditionally synthesized using bases and iodides as promoters (Scheme 1a).^11^ From the academic and industrial view points, alternative reaction media are currently of considerable interest given an increasing emphasis on making the reaction process “greener”, for example, by minimizing the use of organic solvents. Water is the obvious leading candidate in this regard because of its low cost, non-flammability, non-toxicity and low environmental concerns.^12^ Pioneering work on synthesis of aryloxyacetamides in pure water has been reported by nitrile hydration reactions of aryloxyacetonitriles. But for these protocols, Ru-, Os-, Rh-phosphane ligand complexes (Scheme 1b) are used,^13–20^ such as [RuCl_2_(η6-*p*-cymene)PR_2_Cl] (R = aryl, heteroaryl or alkyl),^21^ [OsCl_2_(η6-*p*-cymene)(PMe_2_OH)]^22^ and [RhCl(1,5-cyclooctadiene){P(NMe_2_)_3_}].^23^ It is hampered by the challenge of manufacturing catalysts, the high cost of the catalysts, and the lack of commercially accessible starting materials. The conversion of nitriles to amides by alkaline solutions of hydrogen peroxide is a well-known preparative procedure.^24^ We believe that a nucleophilic substitution between arylboronic acids, which have the advantages of structural diversity, low toxicity, easy availability, greater stability in pure water and can be easily converted into corresponding phenols by oxidative hydroxylation,^25,26^ with 2-bromoacetonitrile could be viable through a careful selection of alkaline solutions of hydrogen peroxide. Herein, we are glad to report an efficient method for the one-pot synthesis of the aryloxyacetamides using arylboronic acids and 2-bromoacetonitrile without the use of a transition metal catalyst or an organic solvent, which is promoted by alkaline solutions of hydrogen peroxide (Scheme 1c).

## Results and discussion

**Fig. 1:**
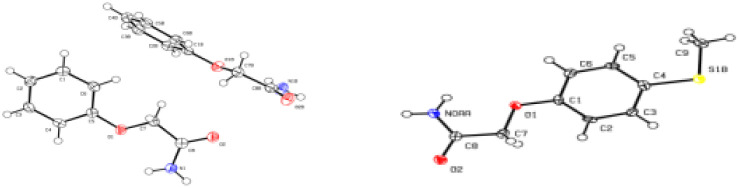
Crystal structure of 3a and 3y (3a: CCDC 2104618; 3y: CCDC 2104616†).

To verify the practicality of this protocol, we commenced to use phenylboric acid (1a) as the model substrate for condition optimization (Table 1). Initially, a series of investigations were conducted to determine the most suitable combination of alkaline solutions of hydrogen peroxide, including KOH, K_2_CO_3_, NaOH, Cs_2_CO_3_, NEt_3_, pyridine solution of hydrogen peroxide (entries 1–6, Table 1). The results showed that the reaction proceeded best when NaOH was used as base to give the product 3a in the yield of 76% (entry 2, Table 1), which stronger base (Cs_2_CO_3_) or weaker bases (K_2_CO_3_, NEt_3_, pyridine) gave a comparably low yield (entries 3–6, Table 1). It should be noted that there was no reaction in the absence of base (entry 7, Table 1). From the results on temperature evaluation, the reaction worked better with the formation of desired product 3a in the yield of 76% when the reaction was carried out at 80 °C (entries 2, 8–10, Table 1). Further optimizations showed that the yield could not be further improved when the reaction time was increased to 6 h (entries 2, 11–12, Table 1). Additionally, the amount of H_2_O_2_ was evaluated, and the results indicated that 0.08 mL (30% aq.) was the most appropriate amount affording the desired product 3a in 76% yield (entries 2, 13–17, Table 1). Finally, it is worth mentioning that the yield of the reaction in nitrogen is inconspicuously different from that in air under the same reaction conditions (entry 18, Table 1) and the structure of 3a was further confirmed by X-ray crystallographic analysis (Fig. 1).

**Table 1:** Initial studies for the reaction of phenylboric acid 1a and 2-bromoacetonitrile^a^

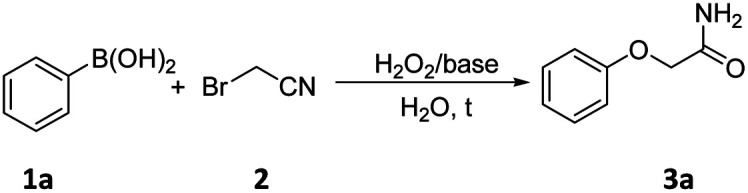					
Entry	Base	30% aq. H_2_O_2_ (mL)	Temperature (°C)	Time (h)	Yield^b^ (%) 3a
1	KOH	0.08	80	4	59
2	NaOH	0.08	80	4	76
3	Cs_2_CO_3_	0.08	80	4	41
4	K_2_CO_3_	0.08	80	4	35
5	NEt_3_	0.08	80	4	40
6	Pyridine	0.08	80	4	Trace
7	—	0.08	80	4	nd^c^
8	NaOH	0.08	40	4	55
9	NaOH	0.08	60	4	64
10	NaOH	0.08	100	4	58
11	NaOH	0.08	80	2	64
12	NaOH	0.08	80	6	75
13	NaOH	—	80	4	nd
14	NaOH	0.06	80	4	40
15	NaOH	0.07	80	4	55
16	NaOH	0.09	80	4	71
17	NaOH	0.10	80	4	67
18	NaOH	0.08	80	4	77^d^

a Reaction conditions: 1a (0.5 mmol), 2 (0.7 mmol), base (1.3 mmol), solvent H_2_O (3 mL), in air.

b Isolated yields based on phenylboric acid 1a.

c nd = not detected the target product.

d Reaction was carried out in nitrogen.

Under the optimized conditions, we subsequently investigated the generality of this one-pot synthesis of aryloxyacetamides from arylboronic acids and 2-bromoacetonitrile promoted by alkaline solutions of hydrogen peroxide as shown in Table 2. Various arylboronic acids containing substituents with an electronic effect and a steric effect could be transformed into the corresponding products 3a–3x in moderate to good yields. Among them, excellent yield of the desired products were obtained in 80–90% when R1 or R2 was substituted with F or Cl group (Table 2, 3b–3j). Then, we evaluated the substitution steric effect of the aromatic ring. Substrates 1m and 1s with steric hindrance were found to be relatively higher reactive for the formation of 3m and 3s. Interestingly, arylboronic acid with sensitive thioether group was also compatible for the reaction under alkaline solutions of hydrogen peroxide obtained 34% yield of the desired product 3y, which was confirmed by X-ray crystallographic analysis (Fig. 1, X-ray of 3y).

**Table 2:** Substrate scope of substituents on the aromatic ring^a^

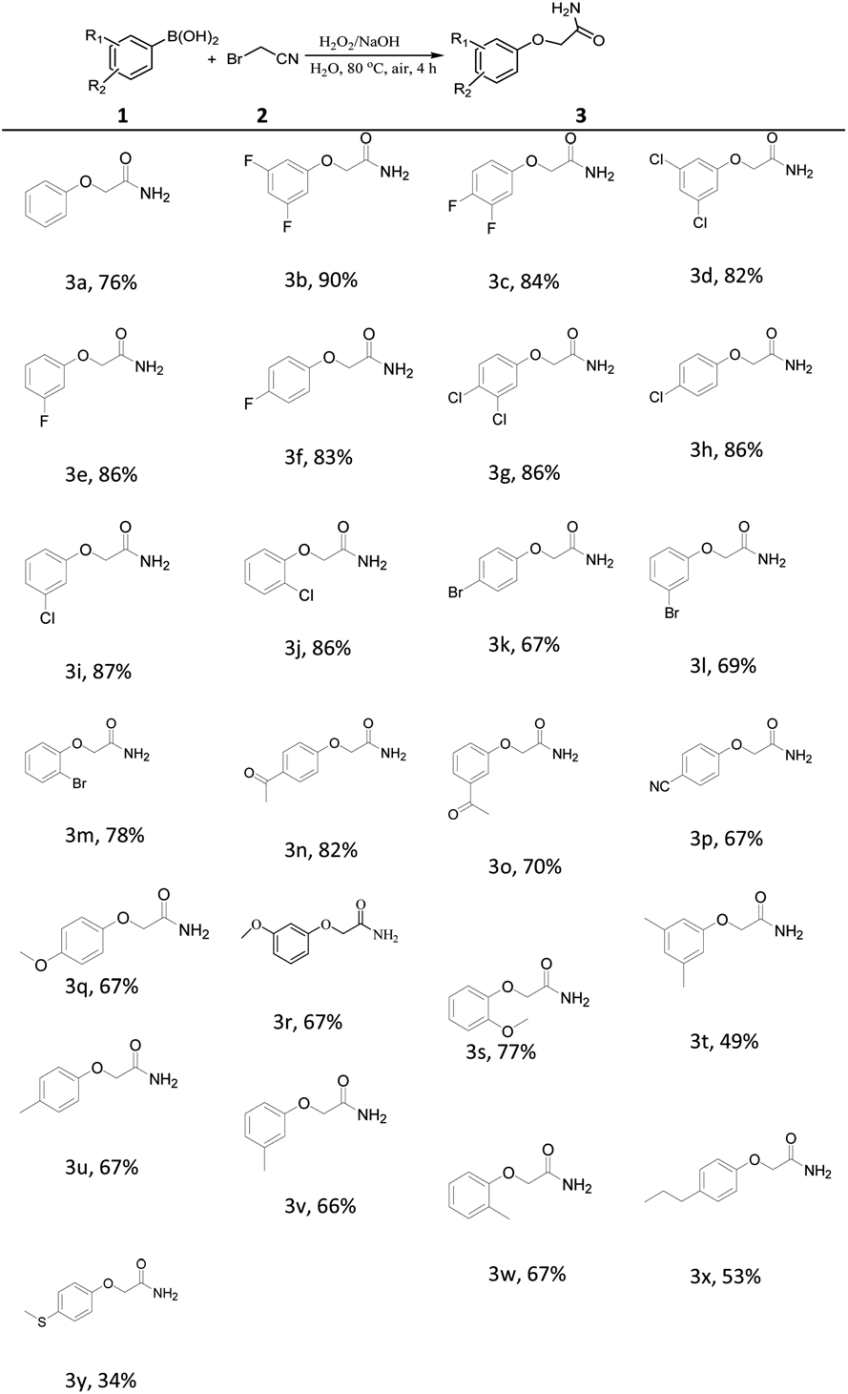

a Reaction conditions: 1 (0.5 mmol), 2 (0.7 mmol), NaOH (1.3 mmol), H_2_O_2_ (30% aq. 0.08 mL), H_2_O (3 mL) at 80 °C under air atmosphere for 4 h.

**Scheme 2:**
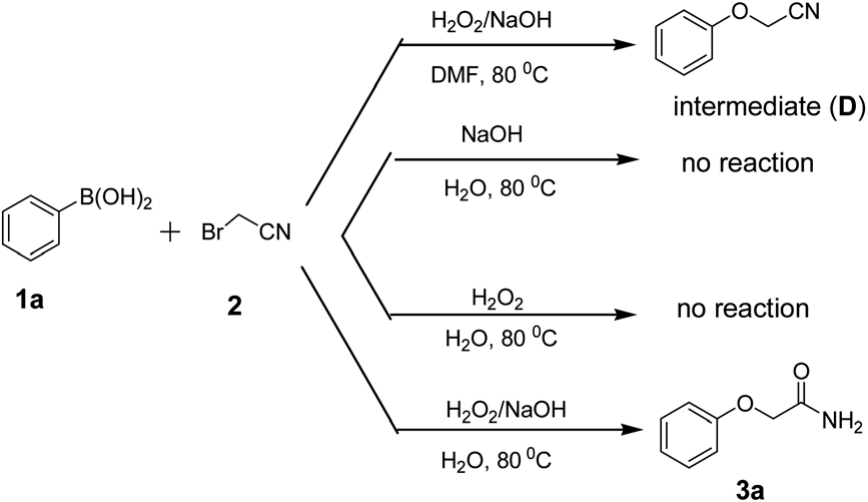
Control experiments and mechanism study.

In order to understand the information on the reaction mechanism, four control experiments were carried out, and the results were listed in Scheme 2. When DMF was used instead of water as solvent, the intermediate phenoxyacetonitrile (D) was obtained in 74% yield. The reaction of 1a with 2 can not take place without H_2_O_2_ or NaOH, and the results demonstrated that the hydrogen peroxide in an alkaline solution was essential for the simultaneous *ipso*-hydroxylation of arylboronic acid and hydration of the nitrile.

**Scheme 3:**
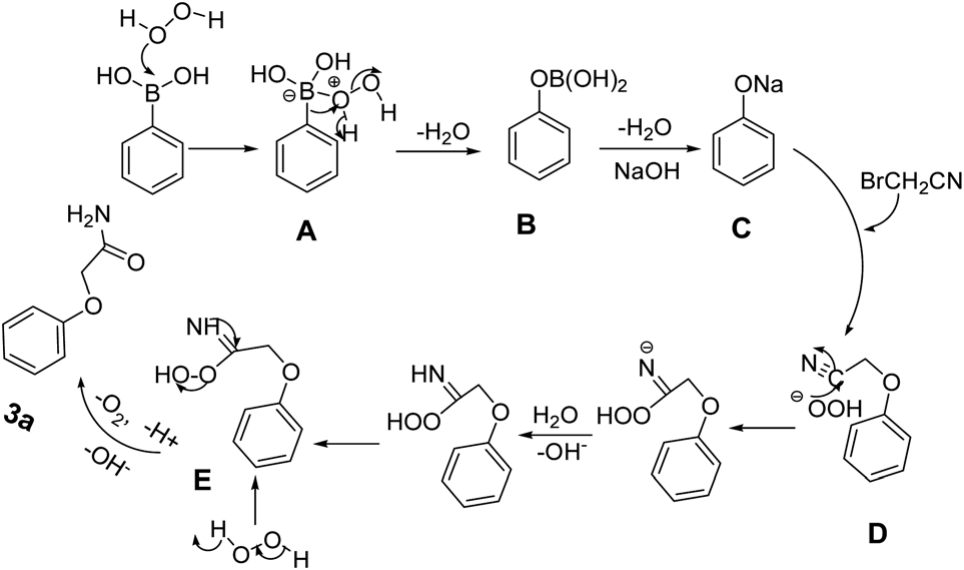
Plausible mechanism for synthesis of aryloxyacetamides.

On the basis of these observations and previous reports,^27,28^ a plausible mechanism for one-pot reaction of arylboronic acids and 2-bromoacetonitrile to synthesize aryloxyacetamides was described as shown in Scheme 3. Initially, arylboric acid reacts with oxidant H_2_O_2_ to form an adduct A which upon rearrangement and subsequent water loss gave adduct B. In the presence of NaOH, hydrolysis of B afforded the sodium phenolate C, which interact with BrCH_2_CN giving rise to intermediate D. Subsequently, the reacting species “HOO–” nucleophilic attacks on the nitrile carbon slowly and efficiently followed by a rapid reaction of the intermediate peroxycarboximidic acid E with hydrogen peroxide to give the amide 3a.

## Conclusions

In conclusion, we have developed a practical synthesis of aryloxyacetamides from the corresponding arylboronic acids and 2-bromoacetonitrile promoted by alkaline solutions of hydrogen peroxide. The primary benefits of this approach include the simultaneous *ipso*-hydroxylation of arylboronic acid and hydration of the nitrile, oxidation-reduction of environmentally beneficial H_2_O_2_, good compatibility with sensitive groups, metal-free and organic solvent-free conditions, and a brief reaction time. A tandem reaction mechanism facilitated by hydrogen peroxide in an alkaline solution is suggested.

## Conflicts of interest

There are no conflicts to declare.

## Supplementary Material

RA-013-D2RA07451F-s001

RA-013-D2RA07451F-s002
