# Supplementary material for: Synthesis of aryloxyacetamides from arylboronic acids and 2-bromoacetonitrile promoted by alkaline solutions of hydrogen peroxide
Source: RSC Adv. 2023 Jan 18;13(4):2631–4. doi: 10.1039/d2ra07451f (PMC9847470; doi:10.1039/d2ra07451f)
Supplement: RA-013-D2RA07451F-s001 [file RA-013-D2RA07451F-s001.pdf]

Electronic Supplementary Material (ESI) for RSC Advances.
This journal is © The Royal Society of Chemistry 2023

Supporting Information

Synthesis of aryloxyacetamides from arylboronic acids and 2-bromoacetonitrile promoted by alkaline solutions of hydrogen peroxide

Mengping Guo,* Yingmin Li, Yongju Wen, Xiuli Shen

Institute of Coordination Catalysis, College of Chemistry and Bio-Engineering, Yichun University, Yichun, Jiangxi 336000, China.

1. General Information

Reactions were monitored by analytical thin-layer chromatography (TLC) on Silica gel plates (GF254). The TLC plates were isualized by shortwave (254 nm) or longwave (365 nm) UV light. Column chromatography was carried out using silica gel (200-300 mesh) to purify the products. Melting points were determined by using a Haineng MP120 melting point apparatus. ^{1}H-NMR and ^{13}C-NMR spectra were recorded with a Bruker Avance II 400 spectrometer (Fallanden, Switzerland) using tetramethylsilane as the internal standard and $CDCl_3$ as the solvent. The high-resolution mass spectras (HRMS) were recorded in Agilent 6210 ESI/TOF mass spectrometer. The crystal structure of the compound was determined by using an Agilent Super Nova CCD Dual X-ray diffractometer. All reagents and solvents were purchased from commercial sources and used without further purification.

2. General procedure for the synthesis of aryloxyacetamides (3a-3y)

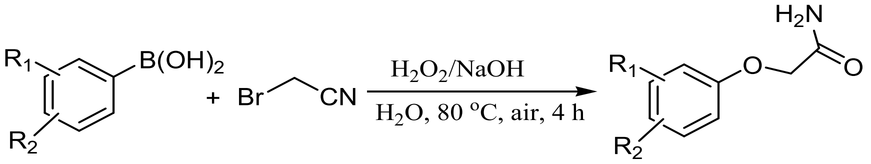

3a-3y

All reactions were carried out under air conditions. A mixture of arylboronic acid (0.5 mmol), 2-bromoacetonitrile (0.7 mmol), base (1.3 mmol) and 3 mL H_2O were taken in an oven dried 10 mL round bottomed flask. To this 30% aq.H_2O_2 0.08

mL was added dropwise and stirred at 80 ^{0}C for 4 h. After completion of the reaction (monitored by TLC) and cooling to ambient temperature, distilled H_2O (10 mL) was added to the mixture, the aqueous layer was extracted with EtOAc (3 × 10 mL). The combined organic layers were dried (Na_2SO_4) and concentrated, and the residue was purified by thin layer chromatography to obtain the desired product by using ethyl acetate/hexane as the eluent.

3. Characterization datas of 3a-3y

3a. 2-Phenoxyacetamide

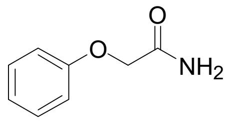

White solid, the yield was 76%, mp 101.1-101.4 oC, 1H NMR (400 MHz, DMSO) δ 7.60 (s, 1H), 7.52 (s, 1H), 7.30 (t, J = 7.4 Hz, 2H), 7.00 – 6.95 (m, 3H), 4.47 (s, 2H); 13C NMR (100 MHz, DMSO) δ 170.36, 157.75, 129.49, 121.18, 114.71, 66.75. Crystallographic data have been deposited at the Cambridge Crystallographic Data Center and allocated with the deposition numbers: CCDC 2104618 for compounds **3a**.

3b. 2-(3, 5-Difluoro-phenoxy)-acetamide

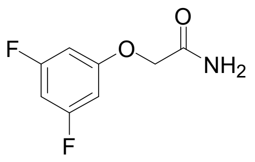

White solid, the yield was 90%, mp 175.5-175.8 ^oC, ^1H NMR (400 MHz, DMSO) δ 7.58 (s, 1H), 7.45 (s, 1H), 7.33 (dt, *J* = 10.5, 9.3 Hz, 1H), 7.06 (ddd, *J* = 12.6, 6.7, 3.0 Hz, 1H), 6.79 (dtd, *J* = 9.0, 3.3, 1.8 Hz, 1H), 4.45 (s, 2H); ^{13}C NMR (100 MHz, DMSO) δ 170.02, 154.78 (dd, J_{C-F} = 9.0, 2.0 Hz), 149.95 (dd, J_{C-F} = 244.9, 13.8 Hz), 144.75 (dd, J_{C-F} = 238.0, 12.7 Hz), 117.92 (d, J_{C-F} = 17.4 Hz), 111.31 (dd, J_{C-F} = 6.1, 3.3 Hz), 104.95 (d, J_{C-F} = 20.4 Hz), 67.75. IR (KBr, cm^{-1}): ν_{N-H} 3474, 3180 cm^{-1}, ν_{C-H} 2925 cm^{-1}(CH_2), $\nu_{C=O}$ 1666 cm^{-1}, $\nu_{C=C}$ 1604, 1517 cm^{-1}, ν_{C-N} 1419 cm^{-1}, $\nu_{=C-O}$ 1259 cm^{-1}, ν_{C-O} 1159 cm^{-1}. HRMS (ESI) m/z: calcd for $C_8H_7F_2NO_2$ $[M + Na]^+$ 210.0337; found 210.0335.

3C. 2-(3,4-Difluoro-phenoxy)-acetamide

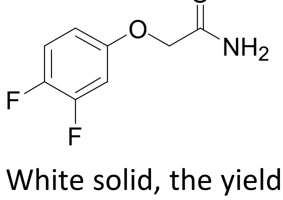

White solid, the yield was 84%, mp 131.1-133.3 ^oC, ^1H NMR (400 MHz, DMSO) δ 7.59 (s, 1H), 7.45 (s, 1H), 6.86 – 6.76 (m, 1H), 6.72 (dd, *J* = 9.4, 2.2 Hz, 2H), 4.49 (s, 2H); ^{13}C

NMR (100 MHz, DMSO) δ 169.67, 164.61 (d, J_{C-F} = 16.2 Hz), 162.19 (d, J_{C-F} = 16.2 Hz), 160.33 (t, J_{C-F} = 14.2 Hz), 99.45 (d, J_{C-F} = 28.6 Hz), 99.45 (d, J_{C-F} = 12.5 Hz), 97.02 (t, J_{C-F} = 26.3 Hz), 67.53. IR (KBr, cm^{-1}): ν $_{N-H}$ 3474, 3180 cm^{-1}, ν $_{C=O}$ 1633 cm^{-1}, ν $_{C=C}$ 1602, 1505, 1477 cm^{-1}, ν $_{C-N}$ 1419 cm^{-1}, ν$_{=C-O}$ 1222 cm^{-1}, ν $_{C-O}$ 1160 cm^{-1}; HRMS (ESI) m/z: calcd for $C_8H_7F_2NO_2$ [M + Na]$^+$ 210.0337; found 210.0339.

3d. 2-(3, 5-Dichloro-phenoxy)-acetamide

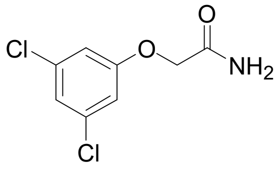

White solid, the yield was 82%, mp 177.2-179.1 ^{o}C, ^{1}H NMR (400 MHz, DMSO) δ 7.58 (s, 1H), 7.43 (s, 1H), 7.13 (s, 1H), 7.03 (s, 2H), 4.51 (s, 2H); ^{13}C NMR (100 MHz, DMSO) δ 169.22, 159.15, 134.47, 120.78, 114.20, 67.00.

3e. 2-(3-Fluoro-phenoxy)-acetamide

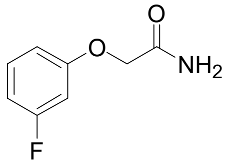

White solid, the yield was 86%, mp 110.6-112.2 ^{o}C, ^{1}H NMR (400 MHz, DMSO) δ 7.58 (s, 1H), 7.44 (s, 1H), 7.38 – 7.27 (m, 1H), 6.88 – 6.71 (m, 3H), 4.47 (s, 2H); ^{13}C NMR (100 MHz, DMSO) δ 170.11, 163.31 (d, J_{C-F} = 242.9 Hz), 159.64 (d, J_{C-F} = 11.2 Hz), 131.13 (d, J_{C-F} = 10.1 Hz), 111.39 (d, J_{C-F} = 2.8 Hz), 108.19 (d, J_{C-F} = 21.1 Hz), 102.85 (d, J = 25.0 Hz), 67.33.

3f. 2-(4-Fluoro-phenoxy)-acetamide

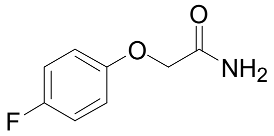

White solid, the yield was 83%, mp 109.9-111.4 ^{o}C,^{1}H NMR (400 MHz, DMSO) δ 7.58 (s, 1H), 7.46 (s, 1H), 7.16 – 7.05 (m, 2H), 7.02 – 6.91 (m, 2H), 4.43 (s, 2H); ^{13}C NMR (100 MHz, DMSO) δ 170.53, 157.28 (d, J = 236.5 Hz), 154.52 (d, J = 1.9 Hz), 116.45 (d, J = 8.1 Hz), 116.22 (d, J = 23.1 Hz), 67.70.

3g. 2-(3,4-Dichloro-phenoxy)-acetamide

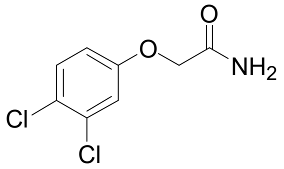

White solid, the yield was 86%, mp 151.5-152.4 °C, ^{1}H NMR (400 MHz, DMSO) δ 7.59 (s, 1H), 7.54 (d, J = 8.9 Hz, 1H), 7.44 (s, 1H), 7.24 (d, *J* = 2.6 Hz, 1H), 6.99 (dd, *J* = 8.9, 2.7 Hz, 1H), 4.50 (s, 2H); ^{13}C NMR (100 MHz, DMSO) δ 169.78, 157.70, 131.92, 131.38, 123.39, 117.29, 116.06, 67.50.

3h. 2-(4-Chloro-phenoxy)-acetamide

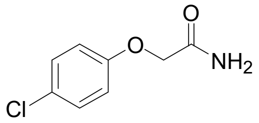

White solid, the yield was 86%, mp 140.5-141.9 °C, ^{1}H NMR (400 MHz, DMSO) δ 7.58 (s, 1H), 7.44 (s, 1H), 7.31 (d, *J* = 8.5 Hz, 2H), 6.97 (d, *J* = 8.4 Hz, 2H), 4.44 (s, 2H); ^{13}C NMR (100 MHz, DMSO) δ 169.83, 156.60, 129.18, 124.91, 116.45, 66.94.

3i. 2-(3-Chloro-phenoxy)-acetamide

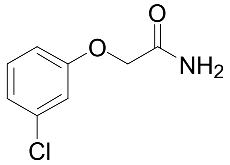

White solid, the yield was 87%, mp 127.4-127.9 °C,^{1}H NMR (400 MHz, DMSO) δ 7.56 (s, 1H), 7.40 (s, 1H), 7.31 (t, *J* = 8.1 Hz, 1H), 7.01 (d, *J* = 8.0 Hz, 2H), 6.92 (d, *J* = 8.4 Hz, 1H), 4.46 (s, 2H); ^{13}C NMR (100 MHz, DMSO) δ 169.61, 158.68, 133.62, 130.86, 121.06, 114.95, 113.67, 66.81.

3j. 2-(2-Chloro-phenoxy)-acetamide

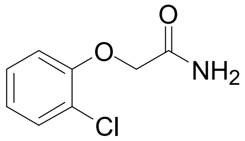

White solid, the yield was 86%, mp149.2-150.1 °C,^{1}H NMR (400 MHz, DMSO) δ 7.54 (s, 1H), 7.41 (d, *J* = 7.9 Hz, 1H), 7.36 (s, 1H), 7.26 (t, *J* = 7.8 Hz, 1H), 7.03 (d, *J* = 8.2 Hz, 1H), 6.96 (t, *J* = 7.6 Hz, 1H), 4.58 (s, 2H); ^{13}C NMR (100 MHz, DMSO) δ 169.54, 153.27, 130.00, 128.18, 122.12, 121.59, 114.12, 67.44.

3k. 2-(4-Bromo-phenoxy)-acetamide

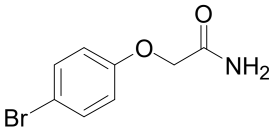

White solid, the yield was 67%, mp 155.0-155.7 °C, ^{1}H NMR (400 MHz, DMSO) δ 7.57 (s, 1H), 7.49 – 7.41 (m, 3H), 6.93 (d, *J* = 9.0 Hz, 2H), 4.44 (s, 2H); ^{13}C NMR (100 MHz, DMSO) δ 170.17, 157.53, 132.55, 117.46, 113.01, 67.28.

3l. 2-(3-Bromo-phenoxy)-acetamide

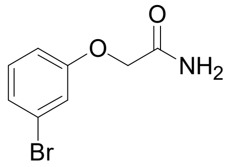

White solid, the yield was 69%, mp 154-155 °C, ^{1}H NMR (400 MHz, DMSO) δ 8.40 (s, 1H), 8.27 (s, 1H), 8.06 (t, *J* = 8.1 Hz, 1H),8.02 – 7.92 (m, 2H), 7.79 (dd, *J* = 8.2, 1.6 Hz, 1H), 5.29 (s, 2H); ^{13}C NMR (100 MHz, MeOD) δ 170.93, 159.97, 132.40, 125.23, 123.24, 119.10, 115.26, 68.09.

3m. 2-(2-Bromo-phenoxy)-acetamide

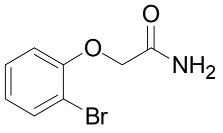

White solid, the yield was 78%, mp 155.1-155.6 °C, ^{1}H NMR (400 MHz, DMSO) δ 7.59 (dd, *J* = 7.9, 1.6 Hz, 1H), 7.51 (s, 1H), 7.33 (ddd, *J* = 8.4, 7.5, 1.6 Hz, 1H), 7.30 (s, 1H), 7.00 (dd, *J* = 8.3, 1.3 Hz, 1H), 6.92 (td, *J* = 7.7, 1.3 Hz, 1H), 4.56 (s, 2H); ^{13}C NMR (100 MHz, DMSO) δ 169.81, 154.55, 133.49, 129.40, 123.10, 114.44, 111.49, 67.87.

3n. 2-(4-Acetyl-phenoxy)-acetamide

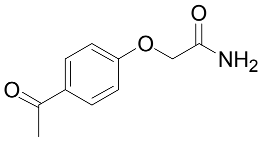

White solid, the yield was 82%, mp 156.1-157.0 °C, ^{1}H NMR (400 MHz, $CDCl_3$) δ 7.99 (d, *J* = 8.9 Hz, 2H), 7.00 (d, *J* = 8.9 Hz, 2H), 6.56 (s, 1H), 6.03 (s, 1H), 4.59 (s, 2H), 2.59 (s, 3H); ^{13}C NMR (100 MHz, $CDCl_3$) δ 196.69, 170.16, 160.72, 130.83, 114.40, 67.04, 26.43. IR(KBr, cm^{-1}): $\nu_{C=O}$ 1710 cm^{-1}(CH_3CO), $\nu_{C=O}$ 1630 cm^{-1} ($CONH_2$), $\nu_{C=C}$ 1600, 1500, 1480 cm^{-1} ,$\nu_{C\text{-}N}$ 1420 cm^{-1}, $\nu_{=C\text{-}O}$ 1220 cm^{-1}, $\nu_{C\text{-}O}$ 1120cm^{-1}. HRMS (ESI) *m/z:* calcd for $C_{10}H_{11}NO_3$ $[M + Na]^+$ 216.0631; found 216.0637.

3o. 2-(3-Acetyl-phenoxy)-acetamide

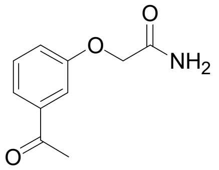

White solid, the yield was 70%, mp 145.7-147.1 °C, ^{1}H NMR (400 MHz, DMSO) δ 7.65 (s, 1H), 7.60 – 7.39 (m, 4H), 7.22 (d, *J* = 6.9 Hz, 1H), 4.53 (s, 2H), 2.55 (s, 3H); ^{13}C NMR (100 MHz, DMSO) δ 198.10, 170.38, 158.35, 138.58, 130.28, 121.81, 120.14, 114.08,

67.19, 27.15.

3p. 2-(4-Cyano-phenoxy)-acetamide

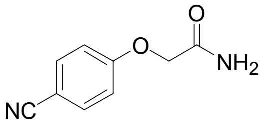

White solid, the yield was 67%, mp 154.5-155.2 ºC, ^{1}H NMR (400 MHz, DMSO) δ 7.73 (t, *J* = 8.8 Hz, 2H), 7.63 (s, 1H), 7.44 (s, 1H), 7.11 (d, *J* = 8.9 Hz, 2H), 4.56 (s, 2H); ^{13}C NMR (100 MHz, DMSO) δ 169.63, 161.68, 134.58, 119.53, 116.25, 103.78, 67.07.

3q. 2-(4-Methoxy-phenoxy)-acetamide

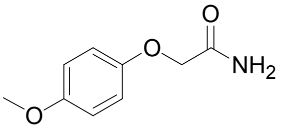

White solid, the yield was 67%, mp 115.3-116.3 ºC, ^{1}H NMR (400 MHz, DMSO) δ 7.51 (s, 1H), 7.41 (s, 1H), 6.94 – 6.83 (m, 4H), 4.36 (s, 2H), 3.69 (s, 3H); ^{13}C NMR (100 MHz, DMSO) δ 170.81, 154.24, 152.24, 116.11, 115.01, 67.88, 55.80.

3r. 2-(3-Methoxy-phenoxy)-acetamide

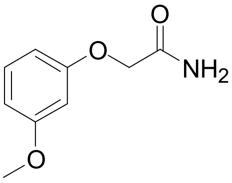

White solid, the yield was 67%, mp 108.7-110.0 ºC, ^{1}H NMR (400 MHz, DMSO) δ 7.55 (s, 1H), 7.45 (s, 1H), 7.20 (t, *J* = 8.1 Hz, 1H), 6.64 – 6.51 (m, 3H), 4.44 (s, 2H), 3.74 (s, 3H); ^{13}C NMR (100 MHz, DMSO) δ170.55, 160.91, 159.42, 130.42, 107.27, 107.22, 101.63, 67.25, 55.51.

3s. 2-(2-Methoxy-phenoxy)-acetamide

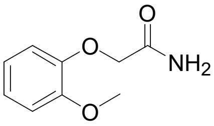

White solid, the yield was 77%, mp 141.7-142.7 ºC, ^{1}H NMR (400 MHz, DMSO) δ 7.44 (s, 1H), 7.33 (s, 1H), 7.02 – 6.85 (m, 4H), 4.42 (s, 2H), 3.77 (s, 3H); ^{13}C NMR (100 MHz, DMSO) δ 170.28, 149.33, 147.41, 122.08, 120.69, 114.71, 112.44, 68.22, 55.55.

3t. 2-(3, 5-Dimethyl-phenoxy)-acetamide

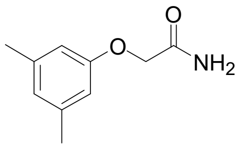

White solid, the yield was 49%, mp 132.8-133.0 ^{o}C, ^{1}H NMR (400 MHz, $CDCl_3$) δ 6.68 (s, 1H), 6.65-6.56 (m, 4H), 4.46 (s, 2H), 2.31 (s, 6H); ^{13}C NMR (10 MHz, $CDCl_3$) δ 171.31, 156.84, 139.22, 123.46, 111.97, 66.64, 20.95. IR (KBr, cm^{-1}): $\nu_{N\text{-}H}$ 3390.2, 3193.5 cm^{-1}, $\nu_{C\text{-}H}$ 2919 cm^{-1}(CH_2), $\nu_{C=O}$ 1680 cm^{-1}, $\nu_{C=C}$ 1613.1, 1594.8, 1509.9, 1474.8 cm^{-1}, $\nu_{C\text{-}N}$ 1409.7 cm^{-1}, $\nu_{=C\text{-}O}$ 1294.9 cm^{-1}, $\nu_{C\text{-}O}$ 1154.1 cm^{-1}; HRMS (ESI) m/z: calcd for $C_{10}H_{13}NO_2$ $[M + Na]^+$ 202.0838; found 202.0839.

3u. 2-p-Tolyloxy-acetamide

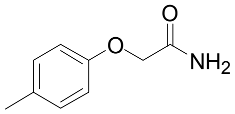

White solid, the yield was 67%, mp 125.8-126.8 ^{o}C, ^{1}H NMR (400 MHz, DMSO) δ 7.48 (s, 1H),7.38 (s, 1H), 7.08 (d, *J* = 8.0 Hz, 2H), 6.84 (d, *J* = 7.8 Hz, 2H), 4.36 (s, 2H), 2.22 (s, 3H); ^{13}C NMR (100 MHz, DMSO) δ 170.18, 155.68, 129.84, 129.79, 114.54, 66.87, 20.07.

3v. 2-m-Tolyloxy-acetamide

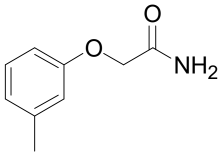

White solid, the yield was 66%, mp 118.9-119.2 ^{o}C, ^{1}H NMR (400 MHz, DMSO) δ 7.53 (s, 1H),7.46 (s, 1H), 7.16 (t, *J* = 7.7 Hz, 1H), 6.79 (d, *J* = 7.1 Hz, 2H), 6.76 (s, 1H), 4.42 (s, 2H), 2.27 (s, 3H); ^{13}C NMR (100 MHz, DMSO) δ 170.26, 157.78, 138.99, 129.19, 121.90, 115.38, 111.66, 66.71, 21.07.

3w. 2-o-Tolyloxy-acetamide

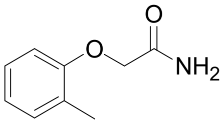

White solid, the yield was 67%, mp 129.2-129.7 ^{o}C, ^{1}H NMR (400 MHz, $CDCl_3$) δ 7.22 – 7.09 (m, 2H), 6.93 (td, *J* = 7.5, 0.7 Hz, 1H), 6.82 – 6.71 (m, 2H), 6.60 (s, 1H), 4.48 (s, 2H), 2.28 (s, 3H); ^{13}C NMR (100 MHz, $CDCl_3$) δ 171.79, 155.39, 131.13, 127.21, 126.54, 121.89, 111.50, 67.25, 16.35.

3x. 2-(4-Propyl-phenoxy)-acetamide

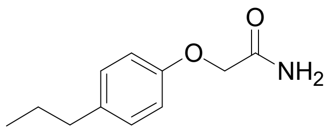

White solid, the yield was 53%, mp 116.3-118.5 °C, ^{1}H NMR (400 MHz, DMSO) δ 7.51 (s, 1H), 7.41 (s, 1H), 7.10 (d, *J* = 8.6 Hz, 2H), 6.96 – 6.84 (m, 2H), 4.39 (s, 2H), 2.57 – 2.38 (m, 2H), 1.53 (dt, *J* = 14.7, 7.4 Hz, 2H), 0.87 (t, *J* = 7.3 Hz, 3H); ^{13}C NMR (100 MHz, DMSO) δ 170.66, 156.32, 135.16, 129.64, 114.91, 67.28, 36.86, 24.78, 14.02. IR (KBr, cm^{-1}): ν_{N-H} 3409, 3183 cm^{-1}, ν_{C-H} 2955, 2868 cm^{-1}(CH_3), ν_{C-H} 2929 cm^{-1}(CH_2), $\nu_{C=O}$ 1680 cm^{-1}, $\nu_{C=C}$ 1585, 1501 cm^{-1}, ν_{C-N} 1428 cm^{-1}, $\nu_{=C-O}$ 1299 cm^{-1}, ν_{C-O} 1177cm^{-1}. HRMS (ESI) *m/z:* calcd for $C_{11}H_{15}NO_2$ [M + Na]$^+$216.0995; found 216.0997.

3y. 2-(4-Methylsulfanyl-phenoxy)-acetamide

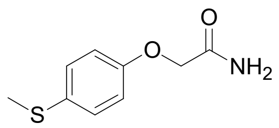

Yellow solid, the yield was 34%, mp 132.3-133.4 °C, ^{1}H NMR (400 MHz, DMSO) δ 7.55 (s, 1H), 7.42 (s, 1H), 7.25 (d, J = 8.8 Hz, 2H), 6.94 (d, J = 8.8 Hz, 2H), 4.42 (s, 2H), 2.42 (s, 3H); ^{13}C NMR (100 MHz, DMSO) δ 170.45, 156.46, 129.62, 129.26, 116.01, 67.31, 16.89. Crystallographic data have been deposited at the Cambridge Crystallographic Data Center and allocated with the deposition numbers: CCDC 2104616 for compounds **3y**.

4. Copies of ^{1}H and ^{13}C NMR spectra of compounds **3a-3y**

4.1. ^{1}H NMR and ^{13}C NMR of 3a

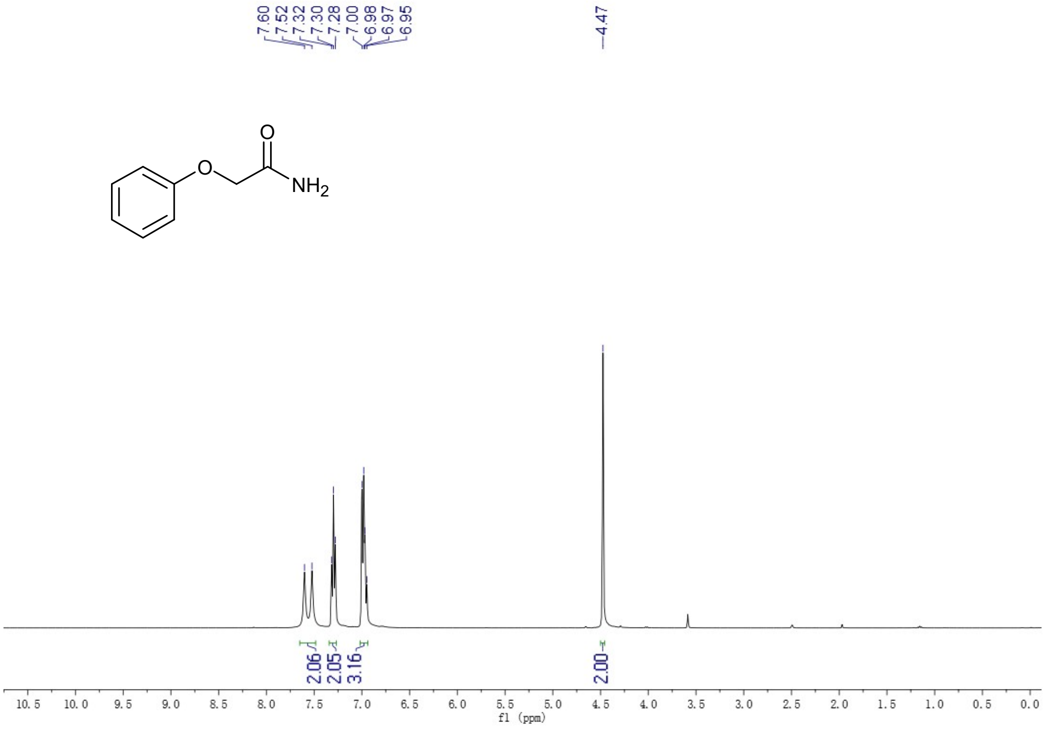

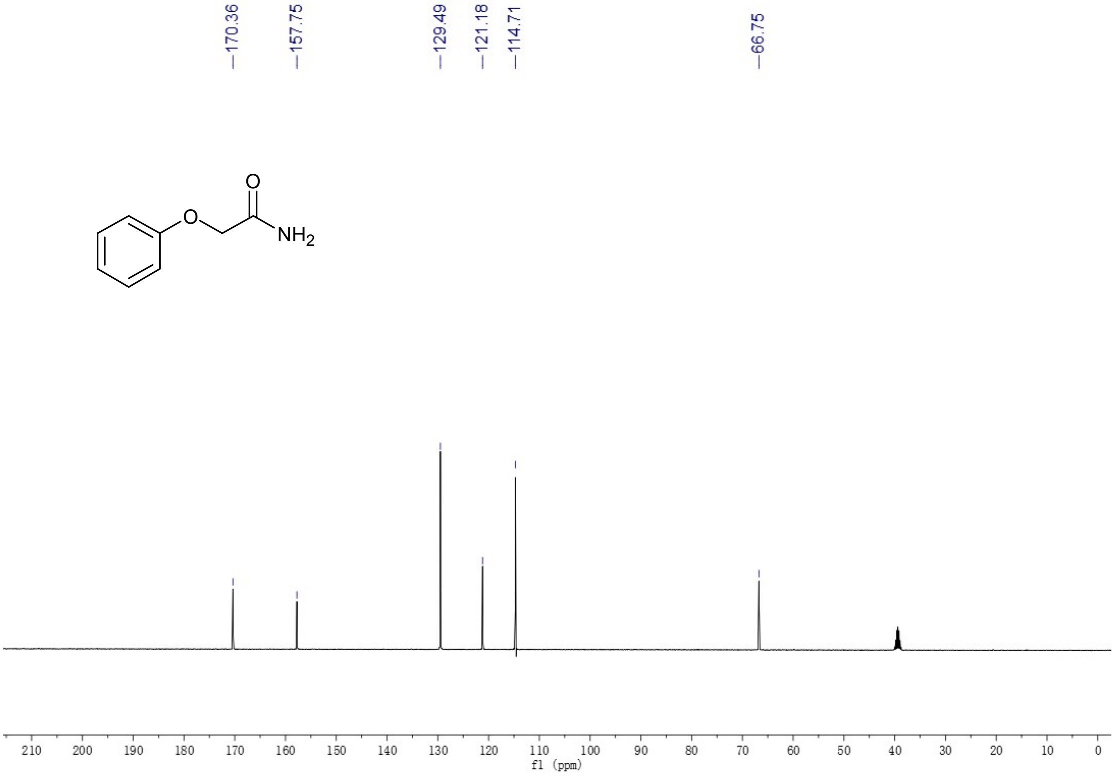

4.2. ^{1}H NMR and ^{13}C NMR of 3b

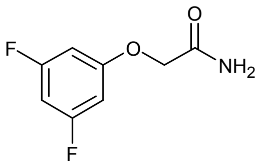

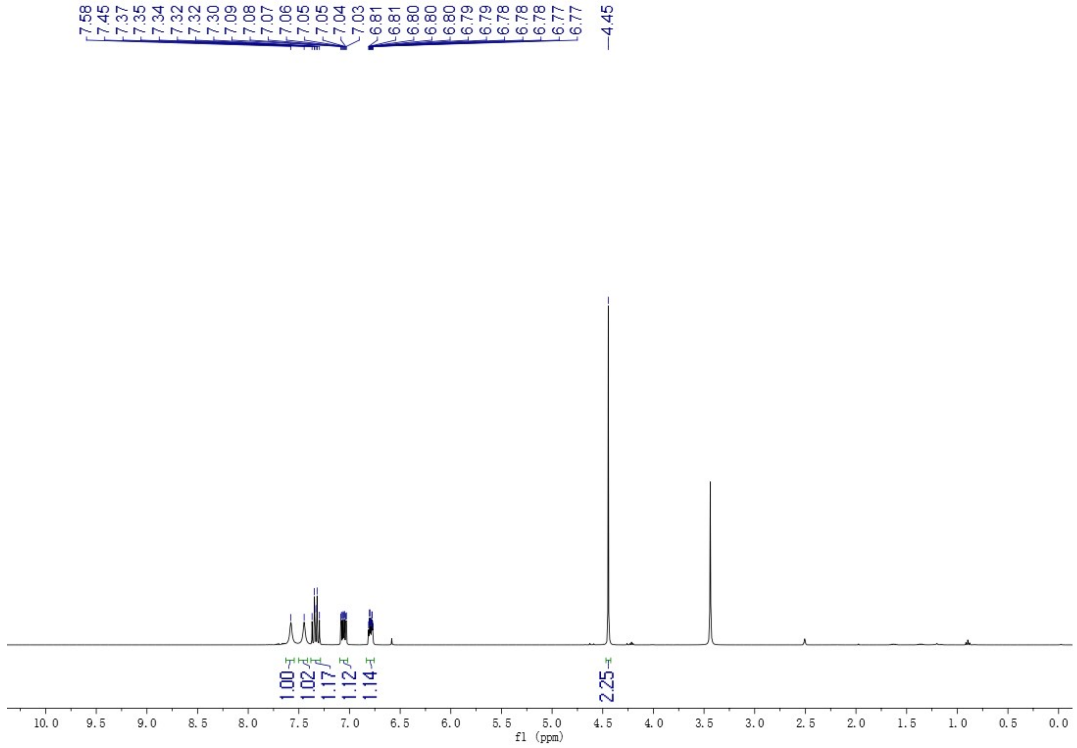

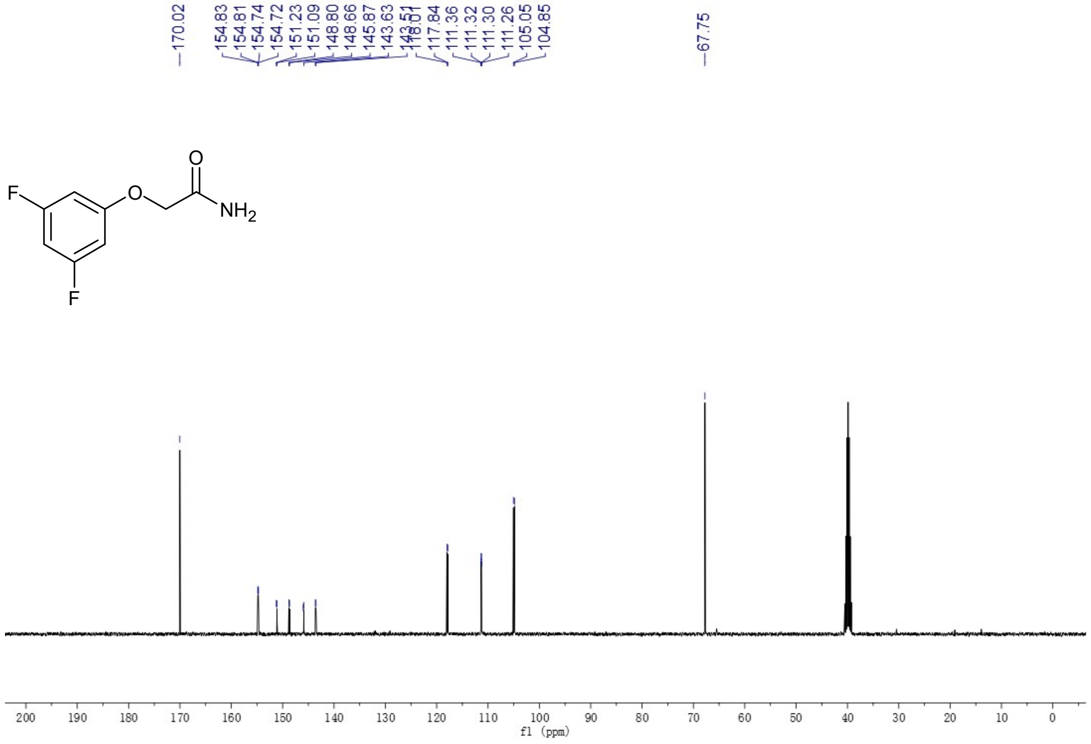

4.3. ^{1}H NMR and ^{13}C NMR of 3c

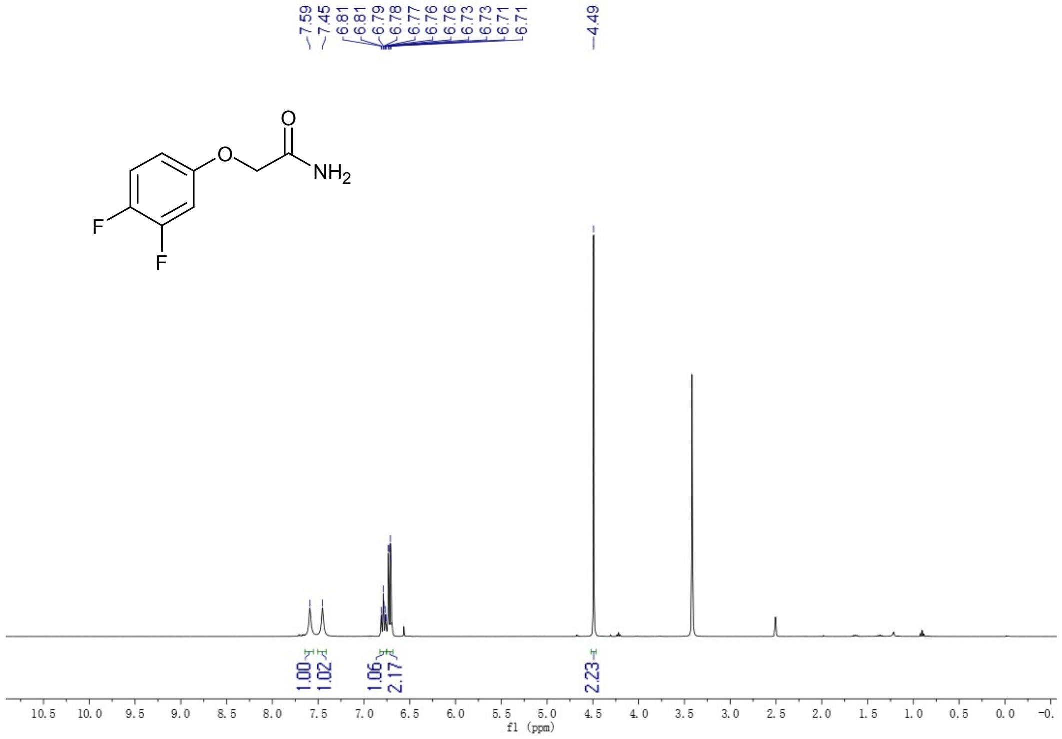

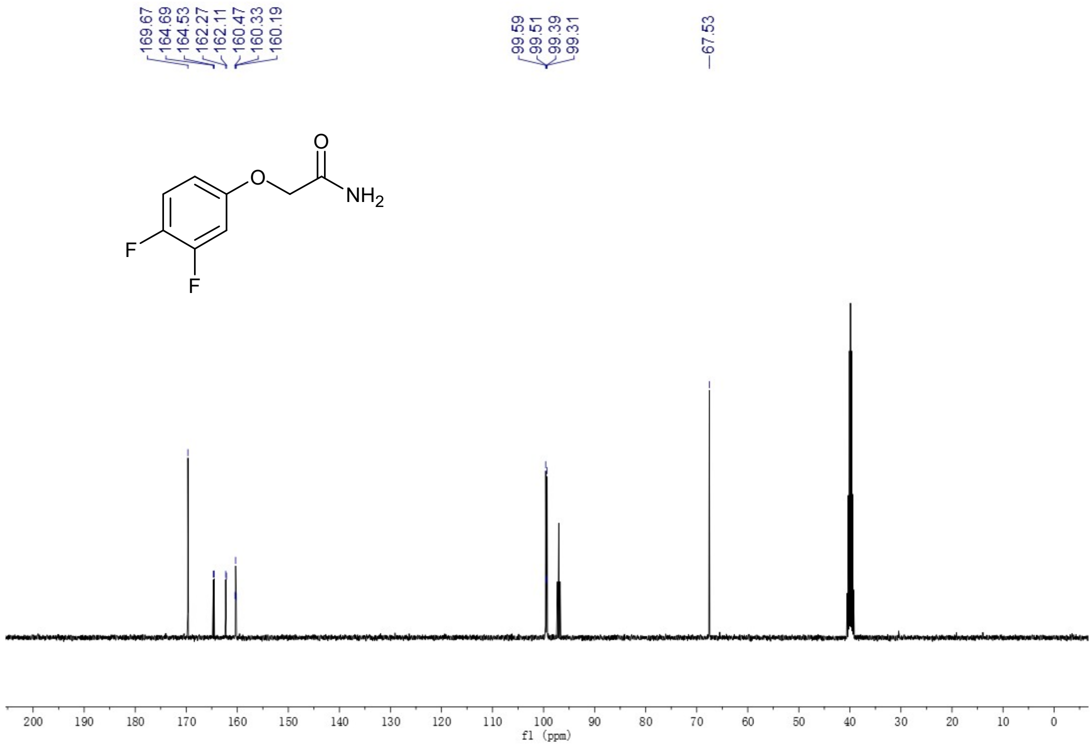

4.4 ^{1}H NMR and ^{13}C NMR of 3d

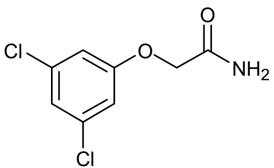

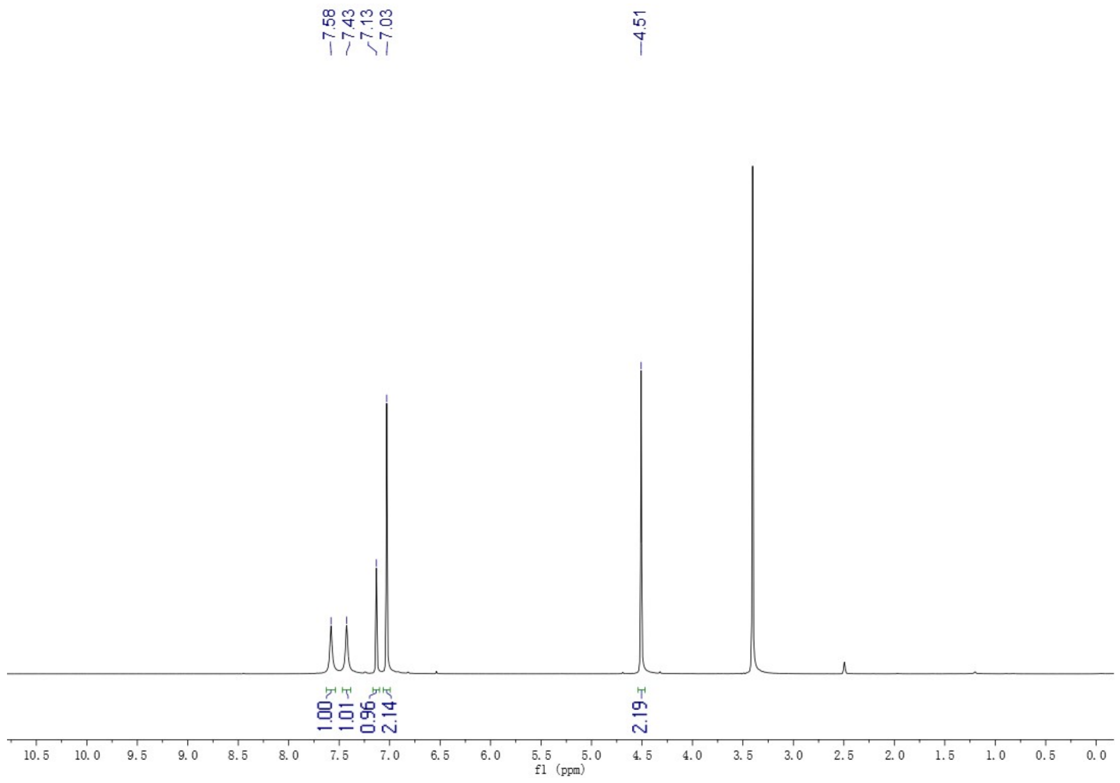

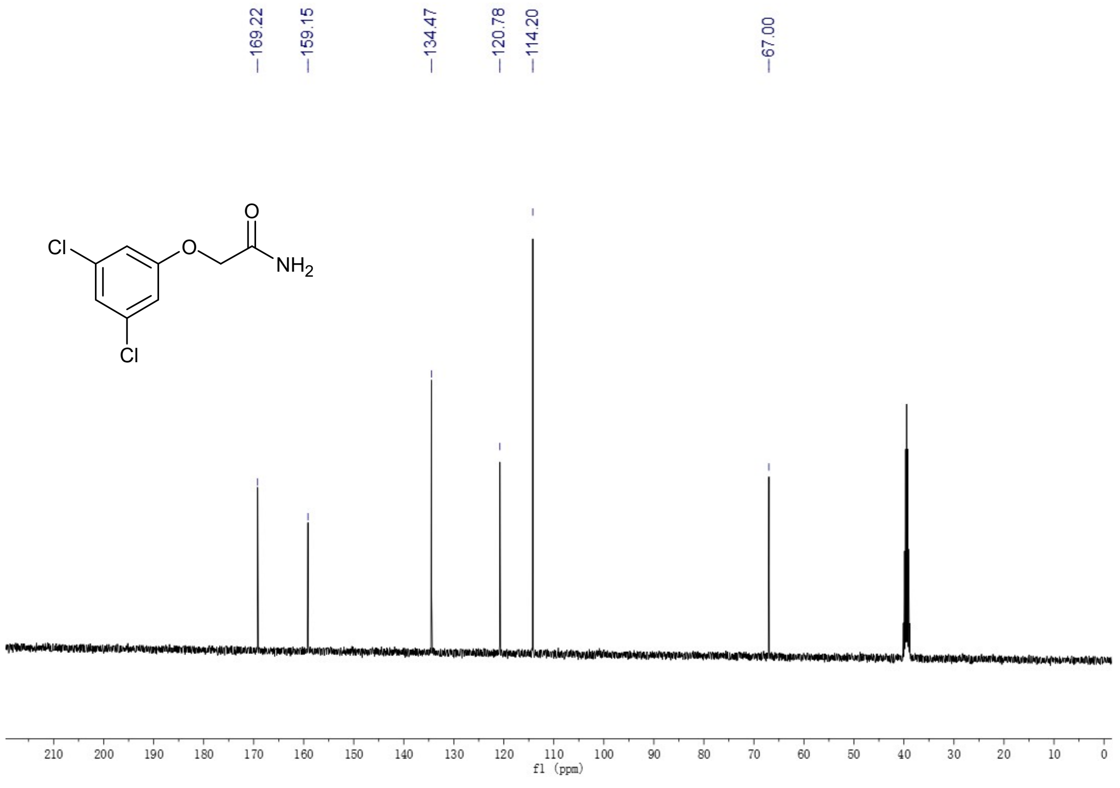

4.5 ^{1}H NMR and ^{13}C NMR of 3e

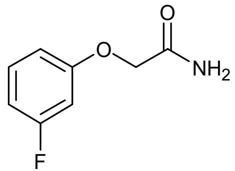

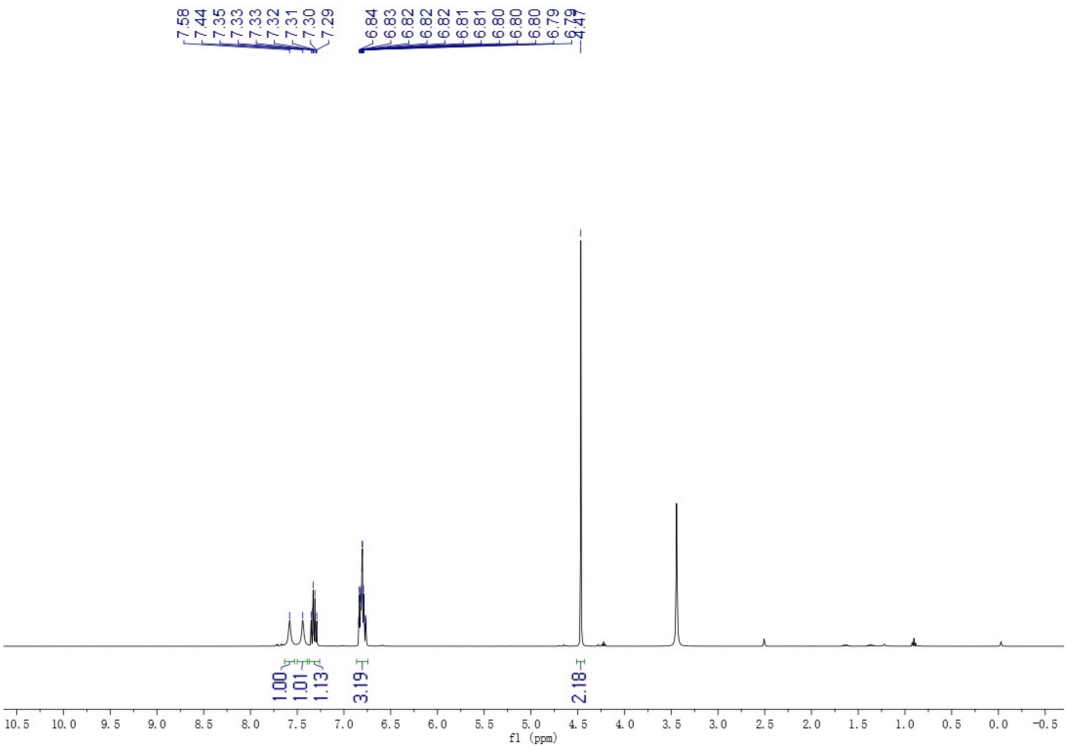

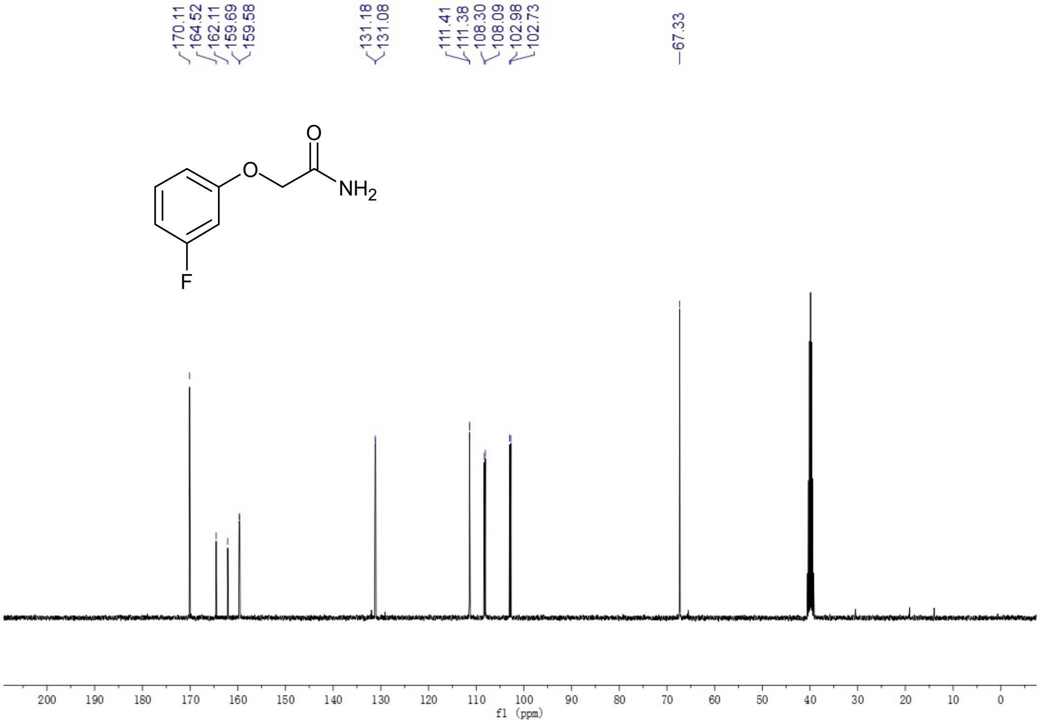

4.6 ^{1}H NMR and ^{13}C NMR of 3f

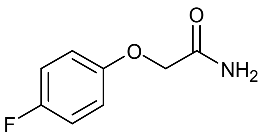

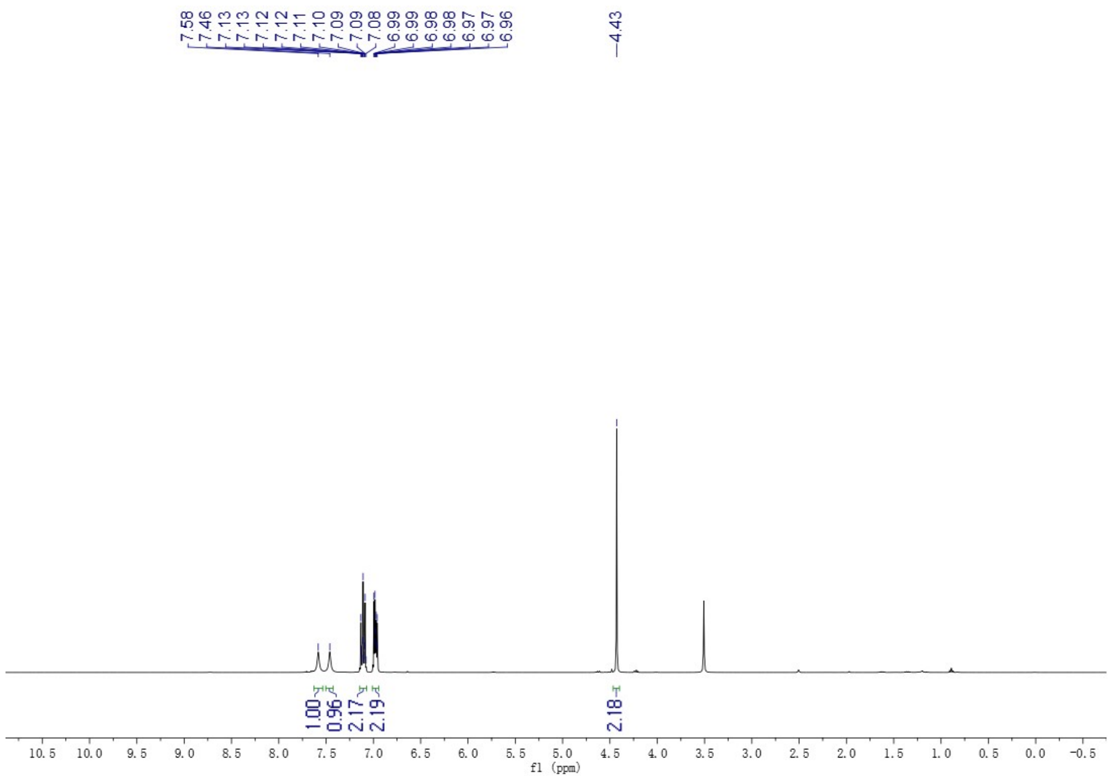

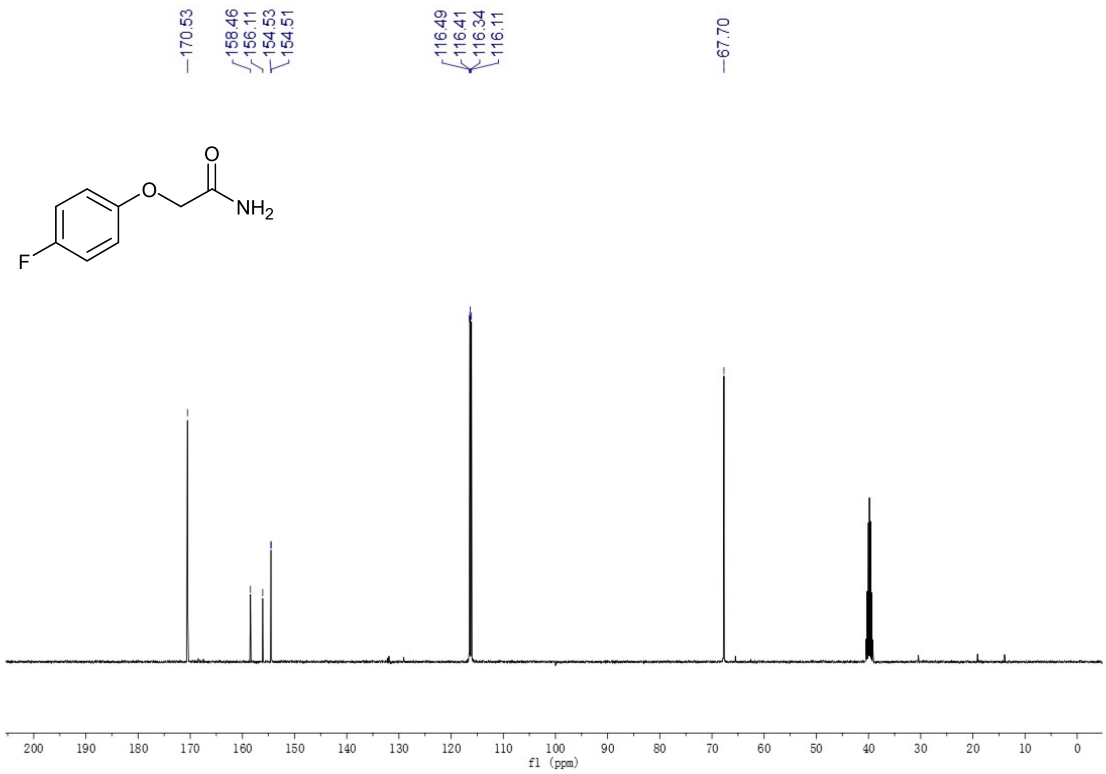

4.7 ^{1}H NMR and ^{13}C NMR of 3g

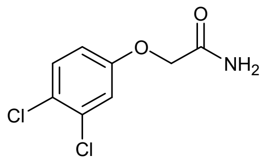

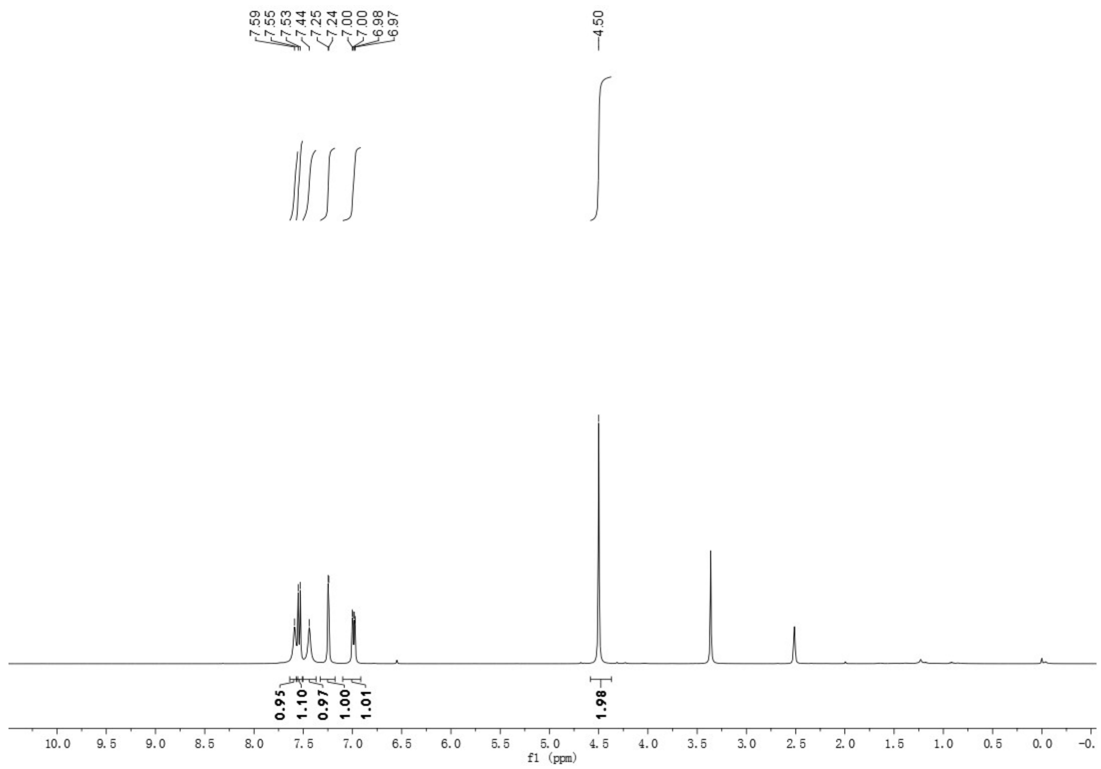

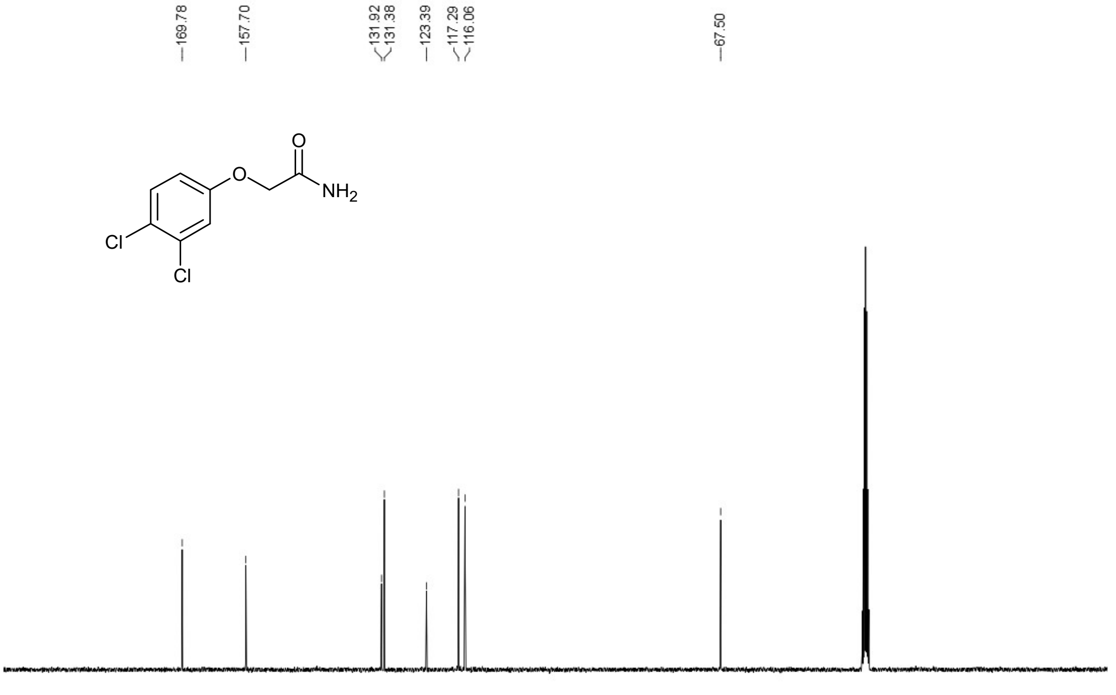

4.8 ^{1}H NMR and ^{13}C NMR of 3h

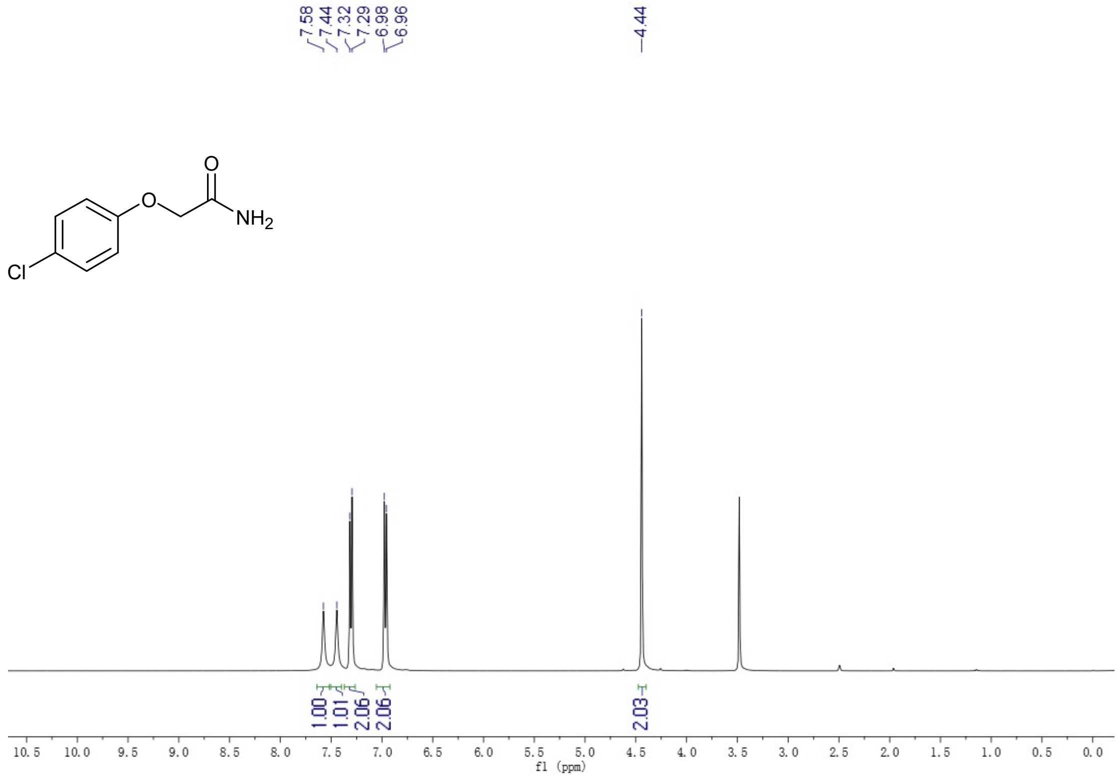

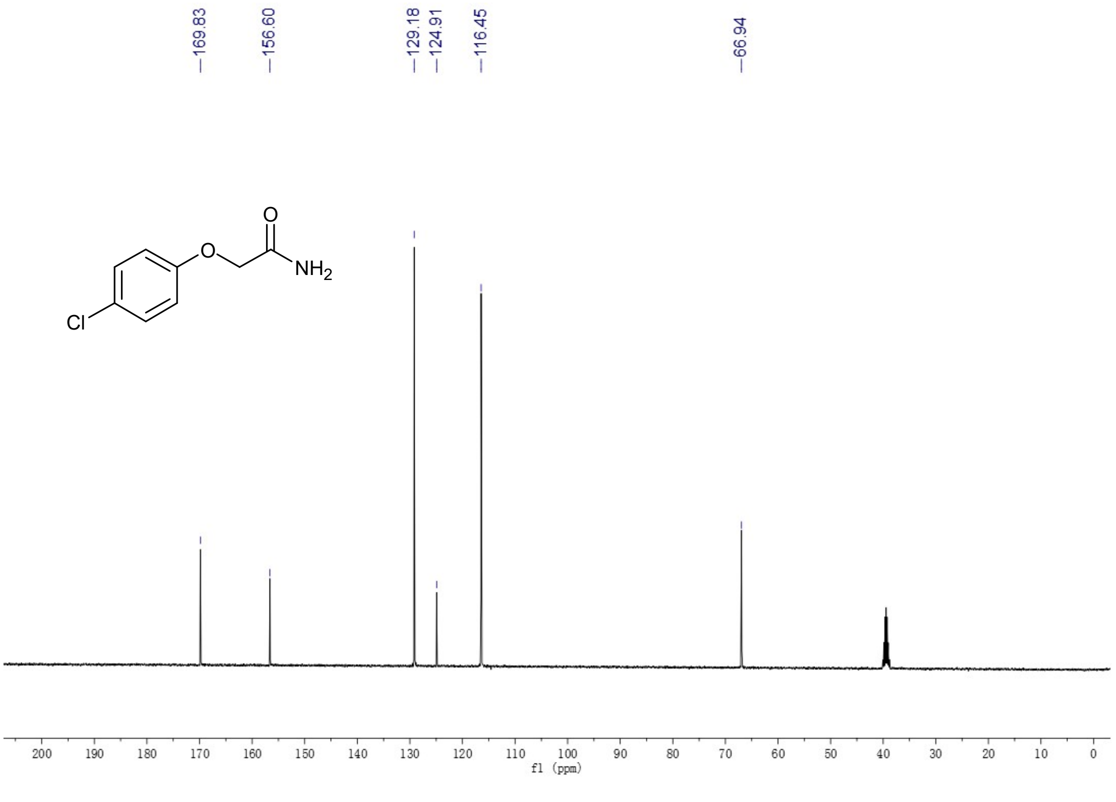

4.9 ^{1}H NMR and ^{13}C NMR of 3i

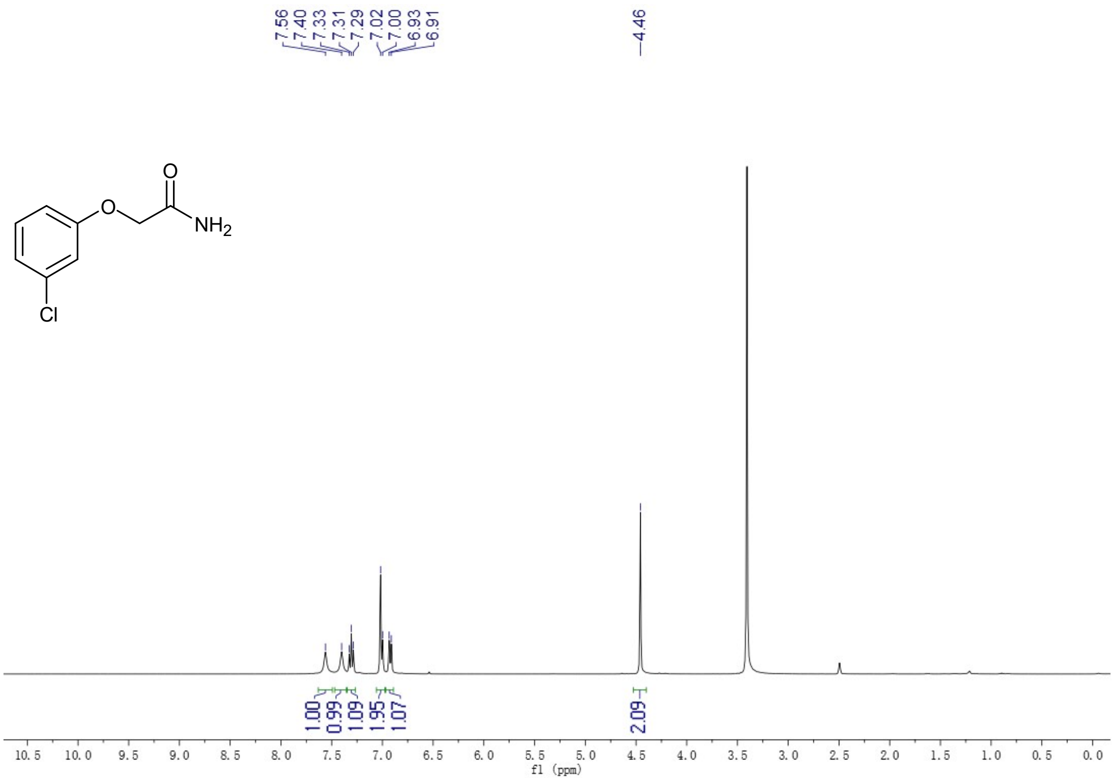

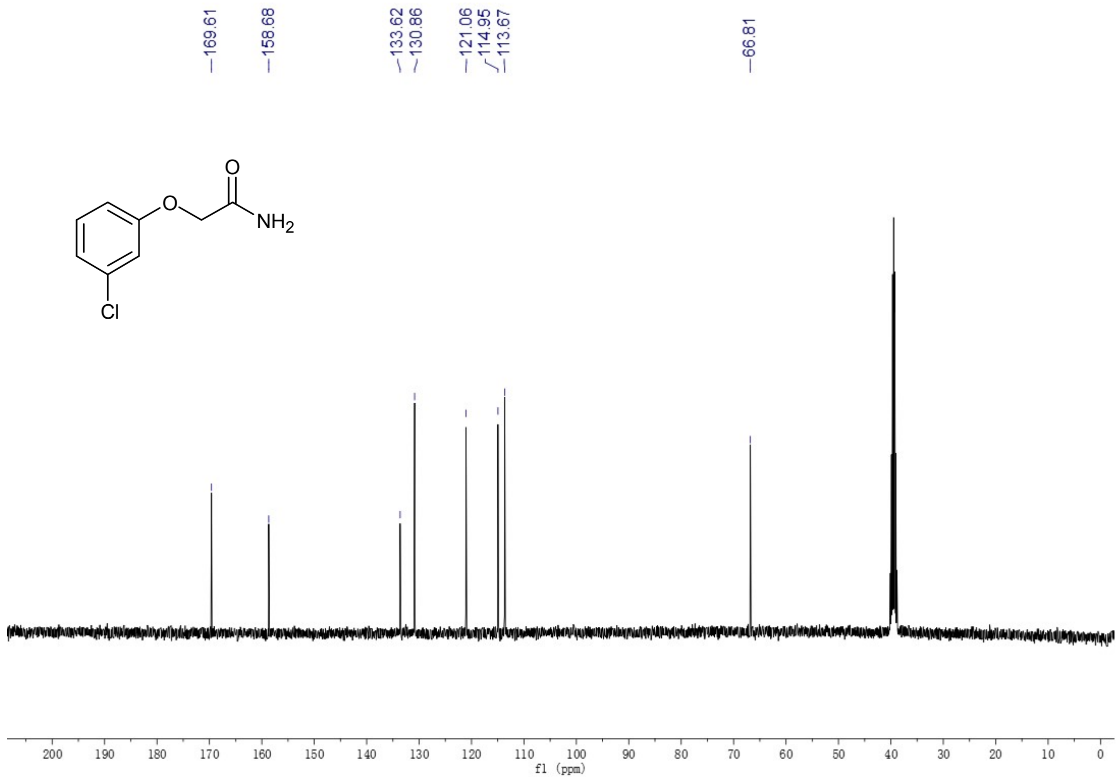

4.10 ^{1}H NMR and ^{13}C NMR of 3j

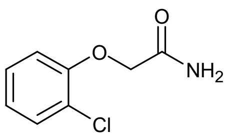

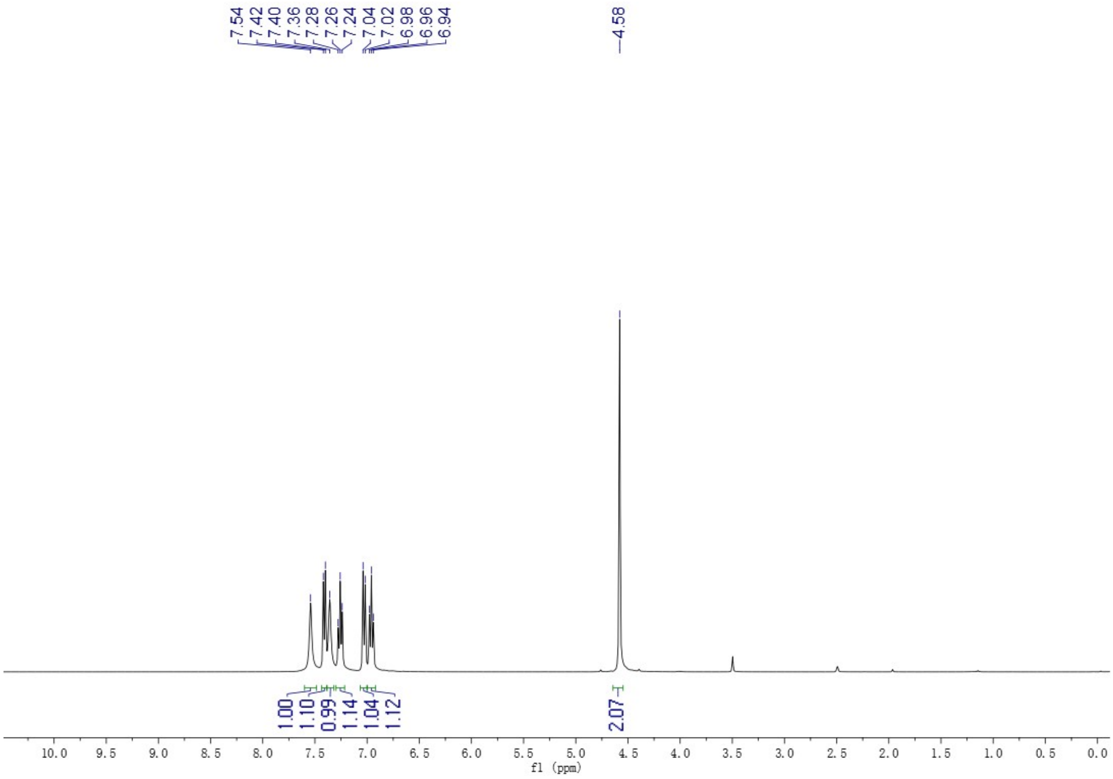

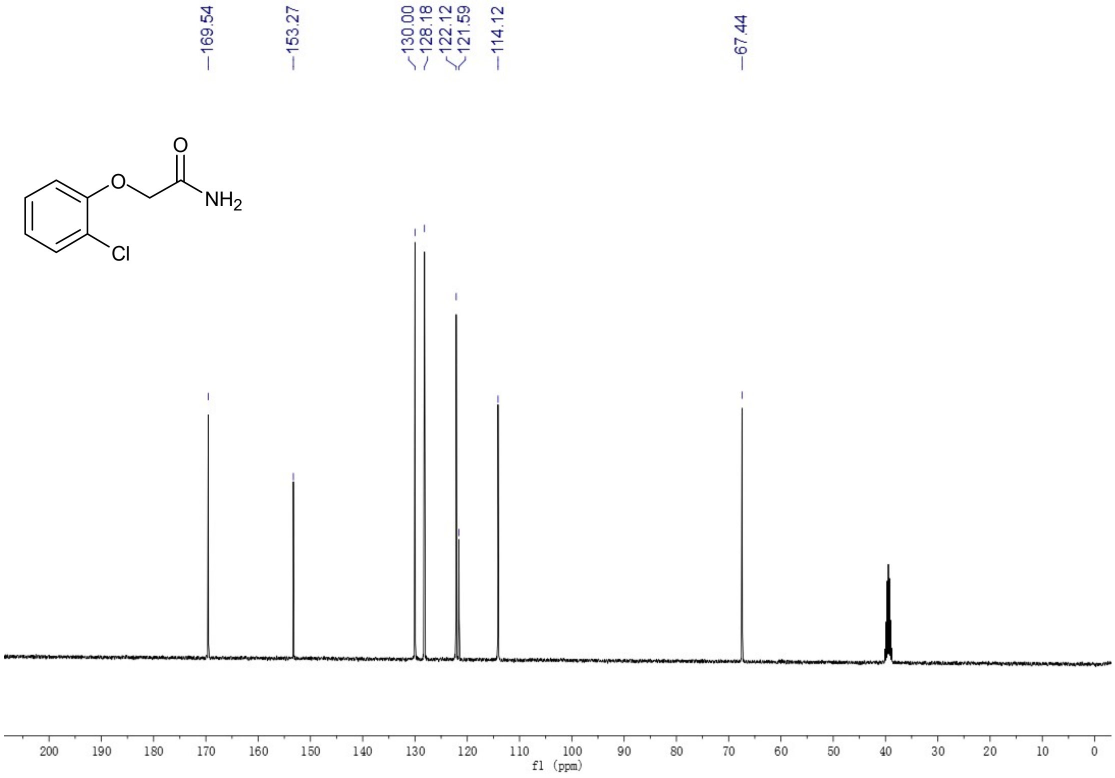

4.11 ^{1}H NMR and ^{13}C NMR of 3k

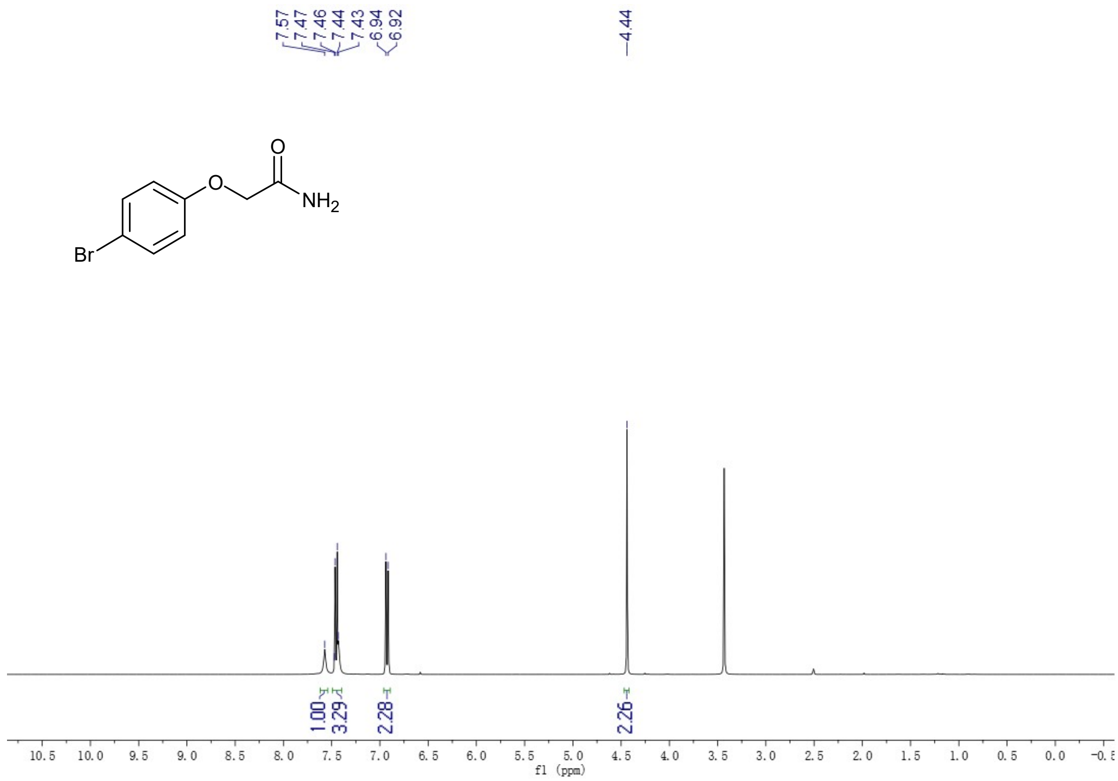

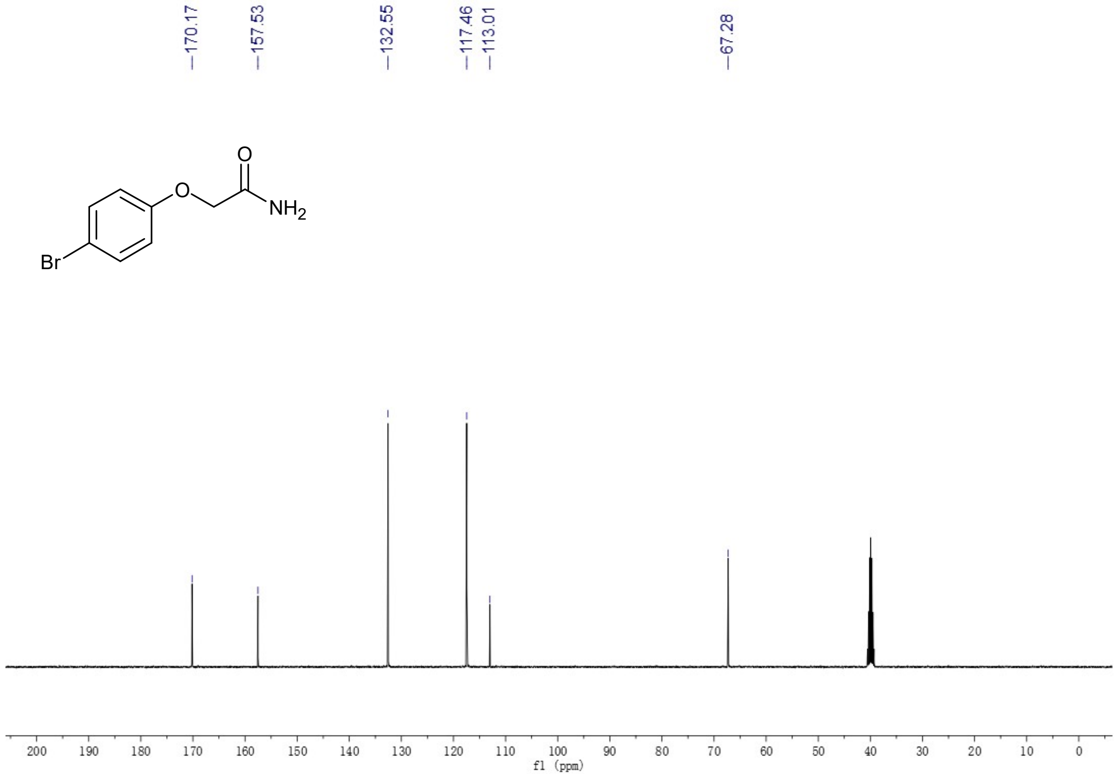

4.12 ^{1}H NMR and ^{13}C NMR of 3l

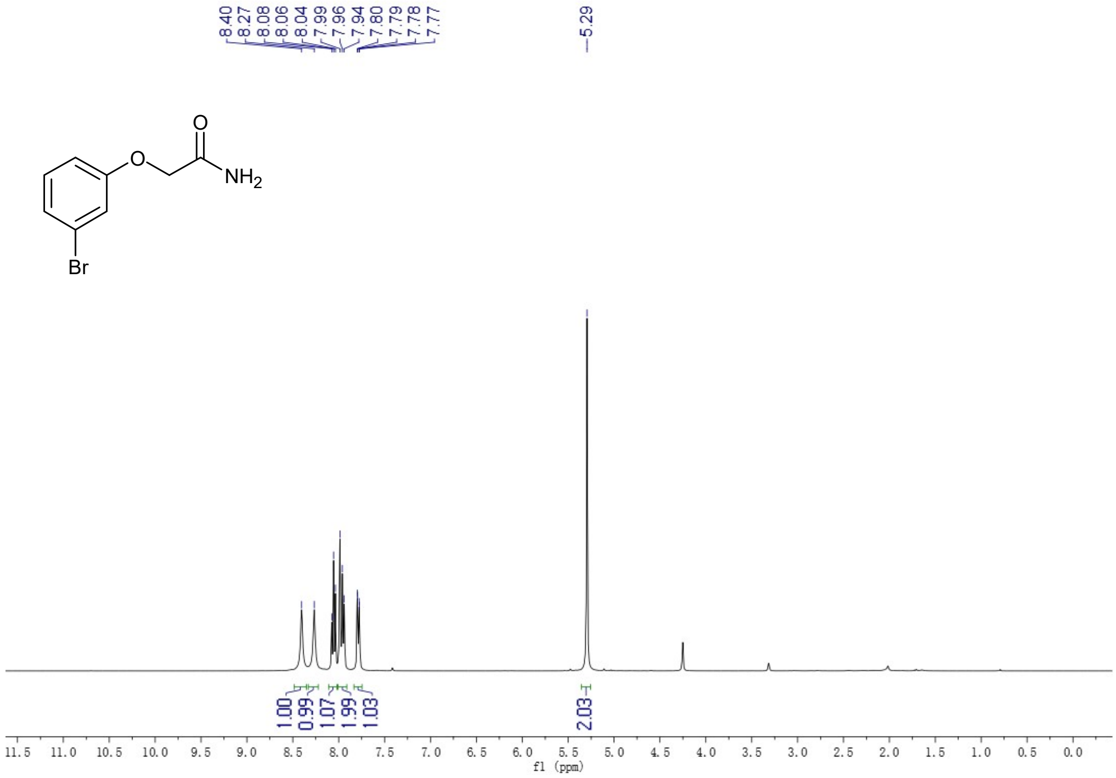

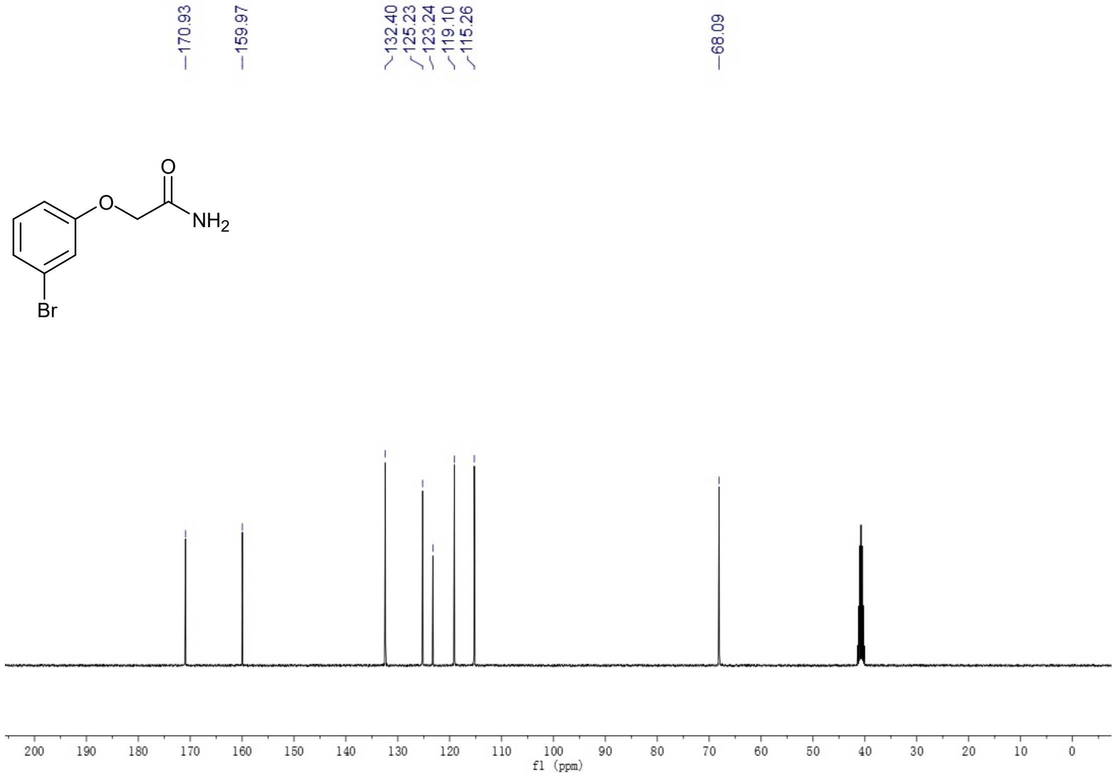

4.13 ^{1}H NMR and ^{13}C NMR of 3m

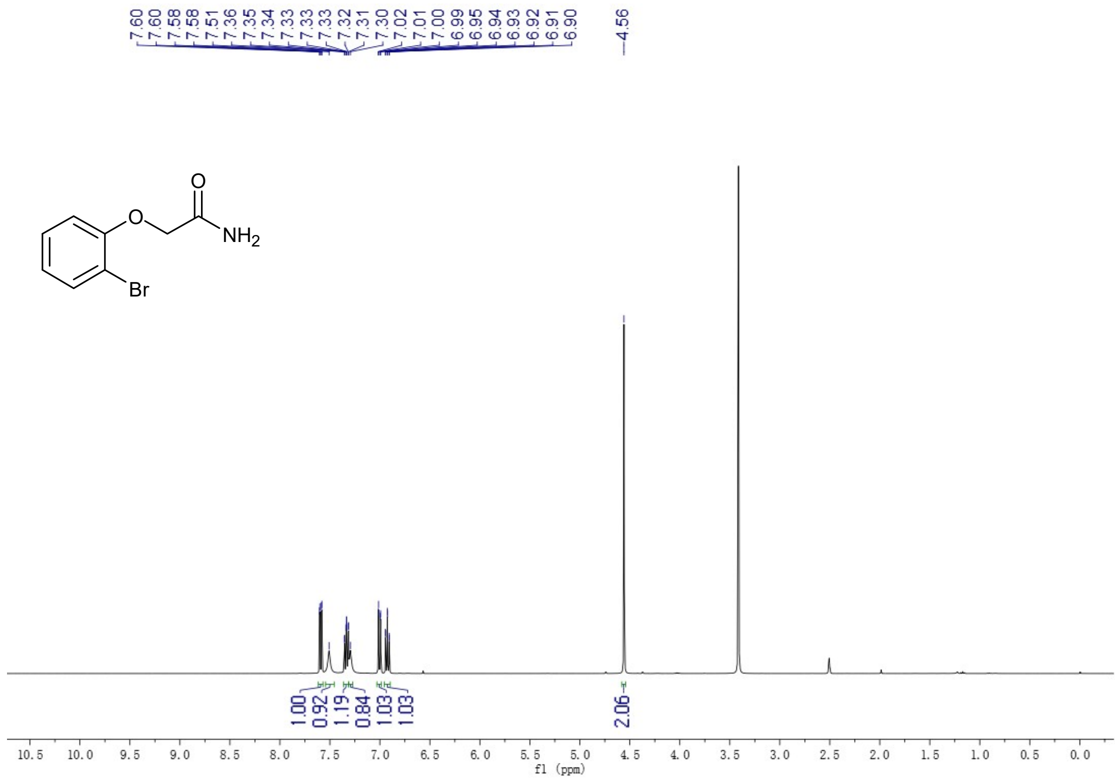

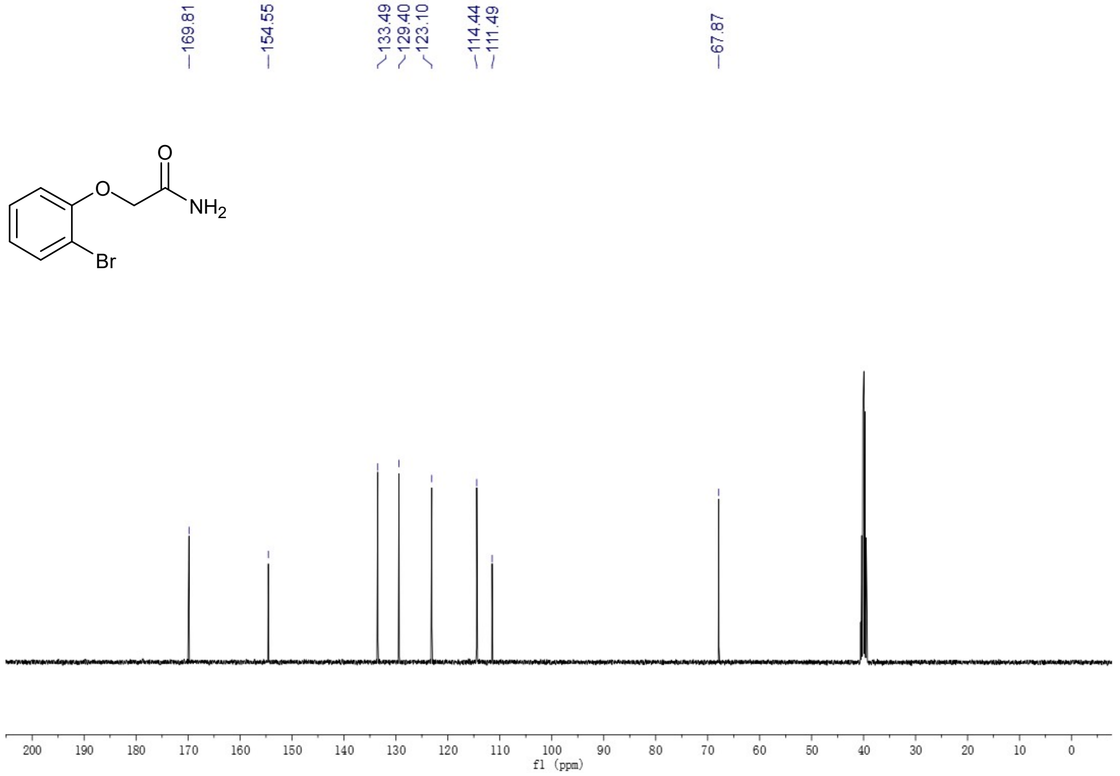

4.14 ^{1}H NMR and ^{13}C NMR of 3n

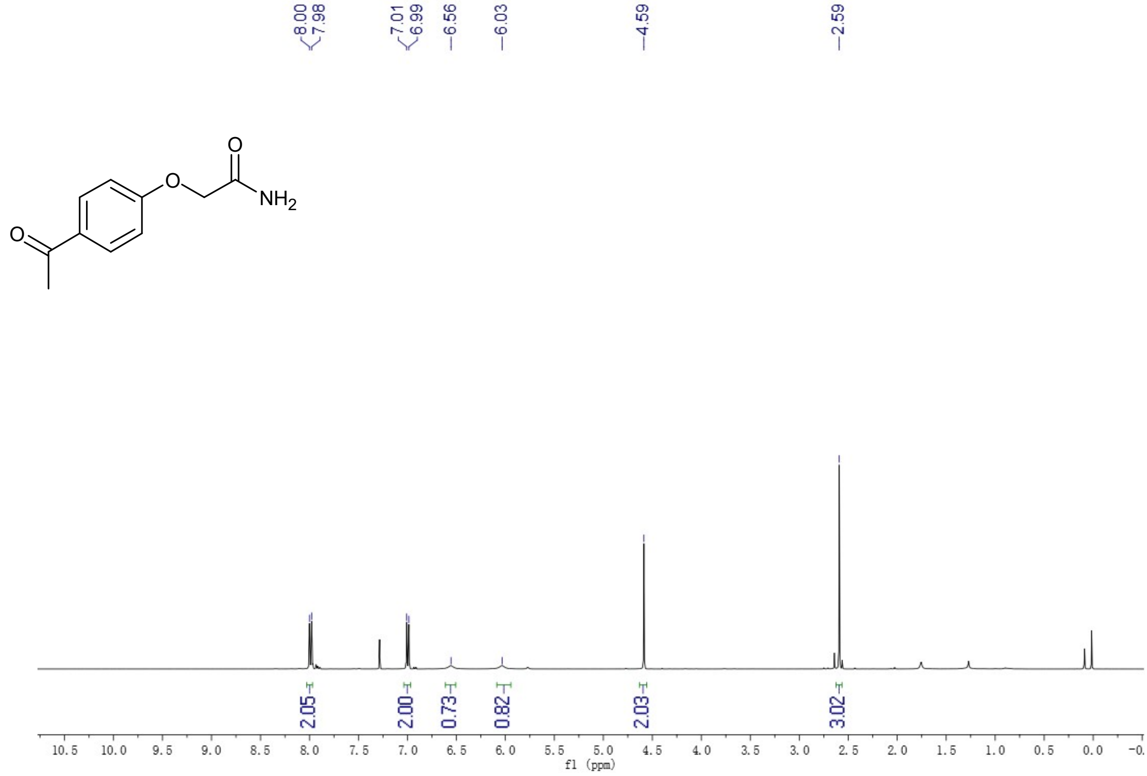

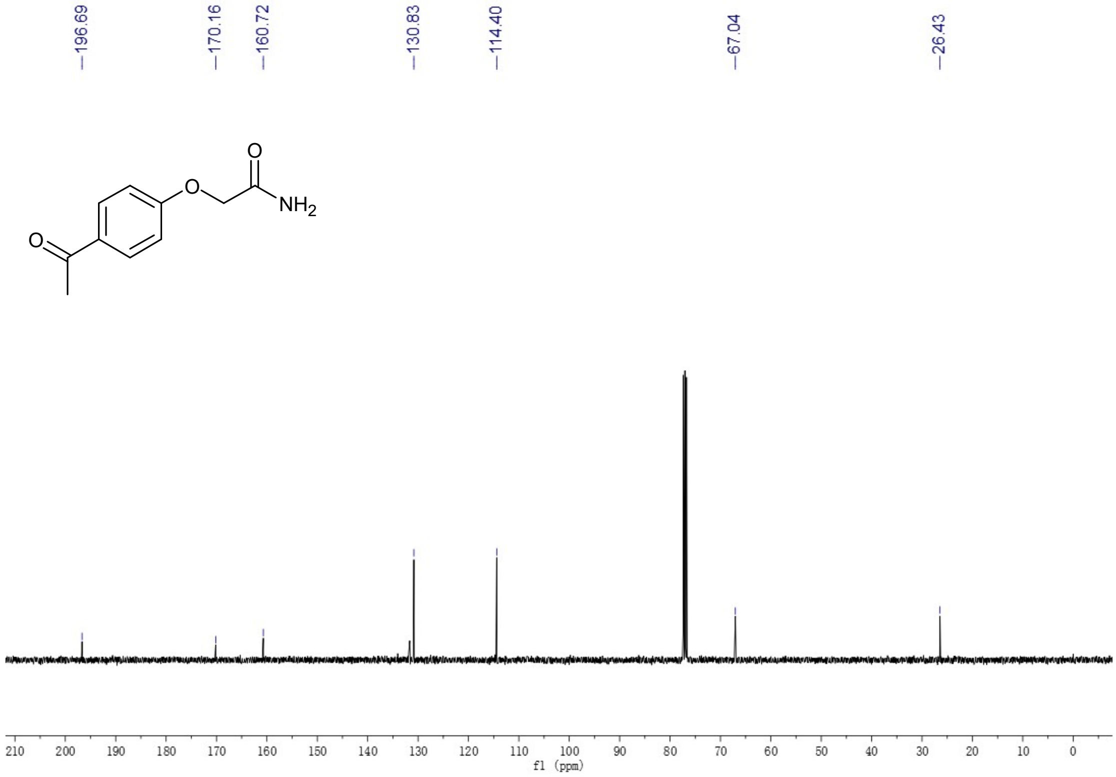

4.15 ^{1}H NMR and ^{13}C NMR of 3o

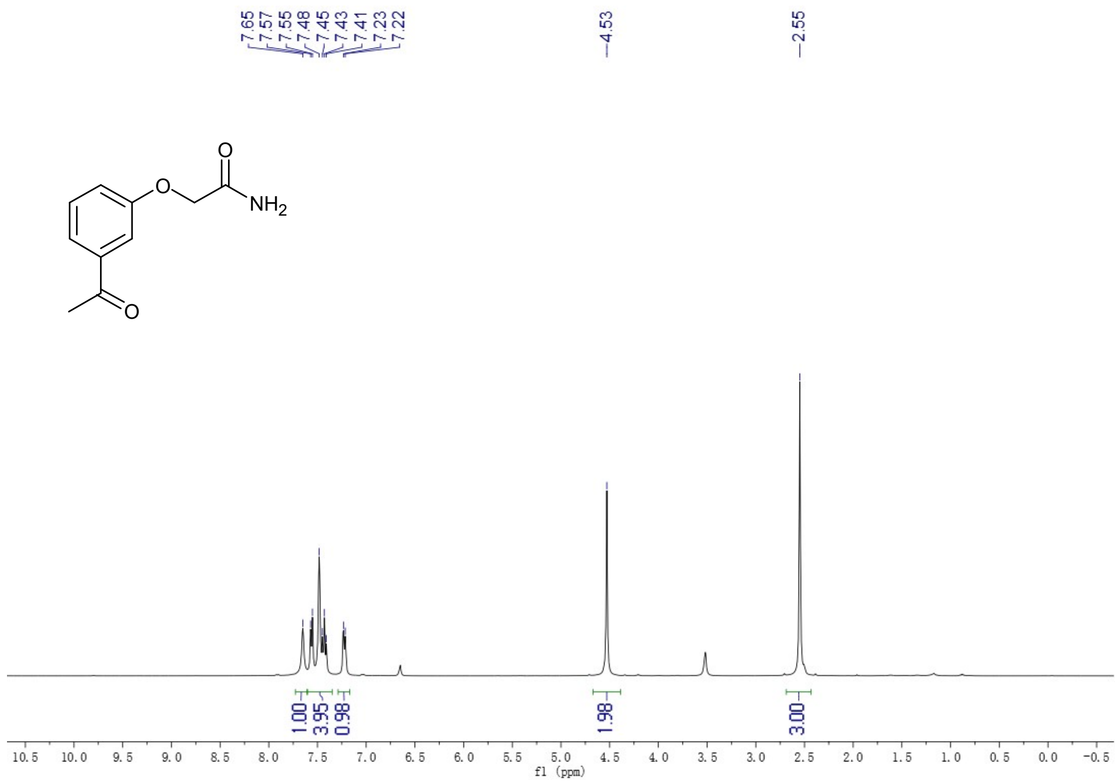

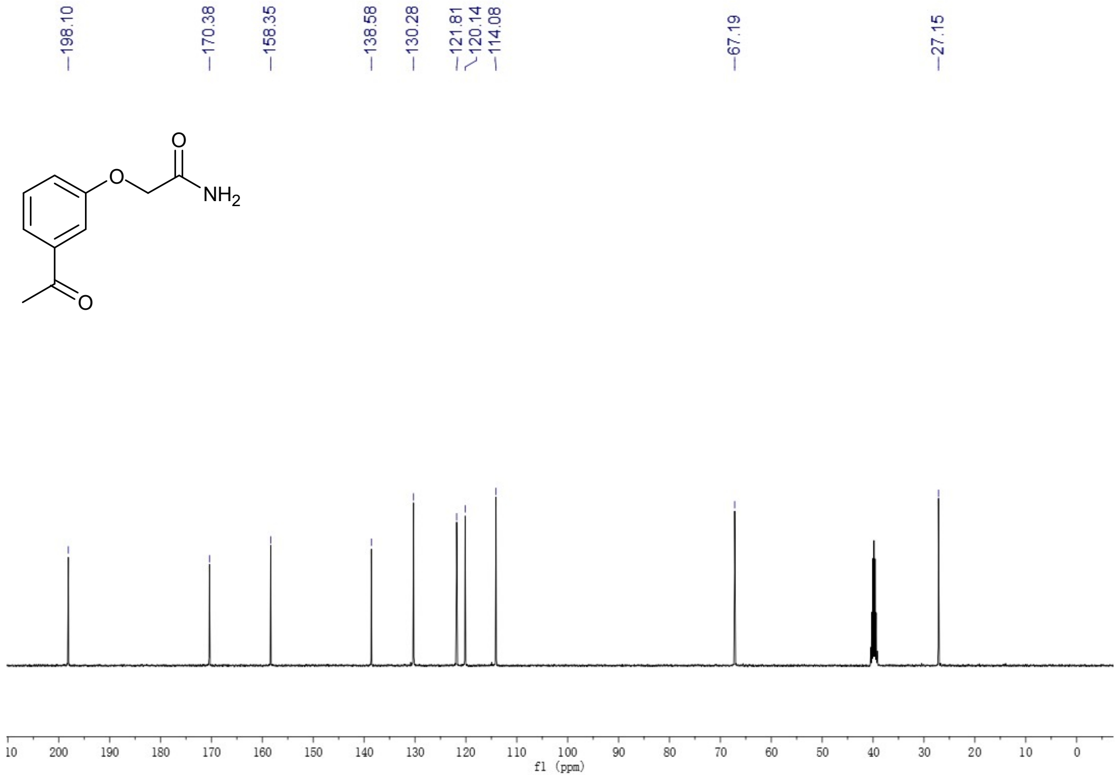

4.16 ^{1}H NMR and ^{13}C NMR of 3p

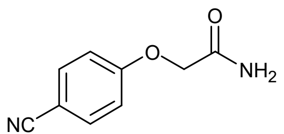

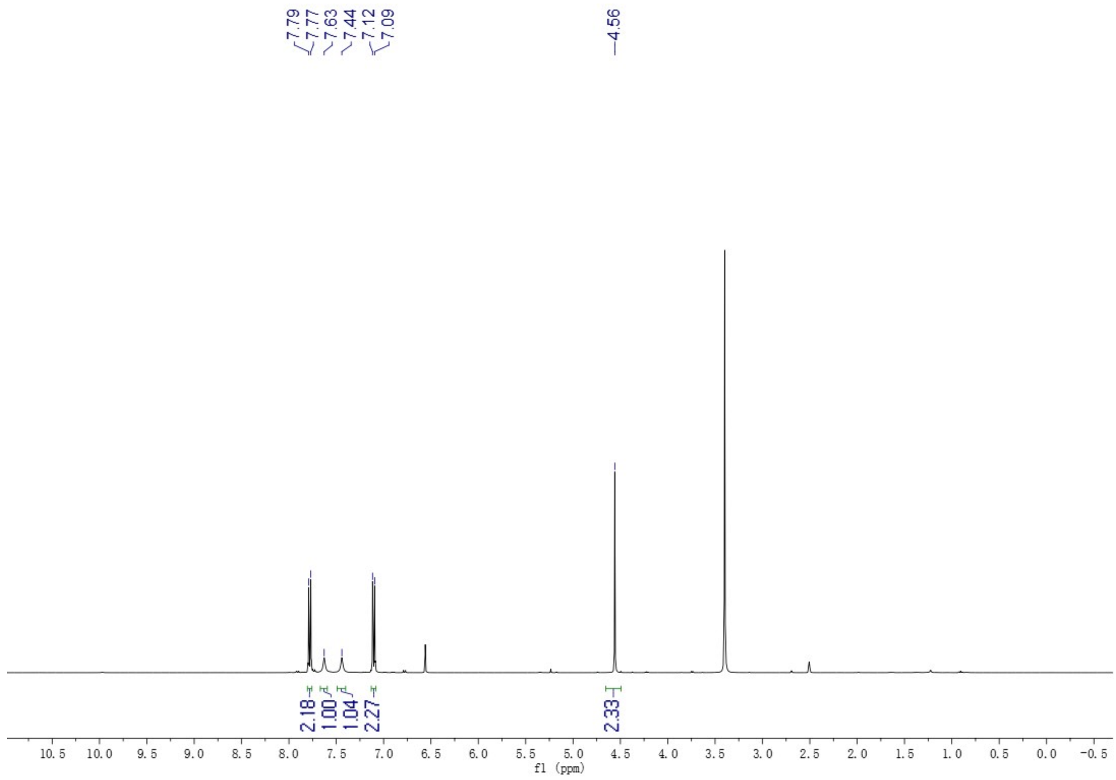

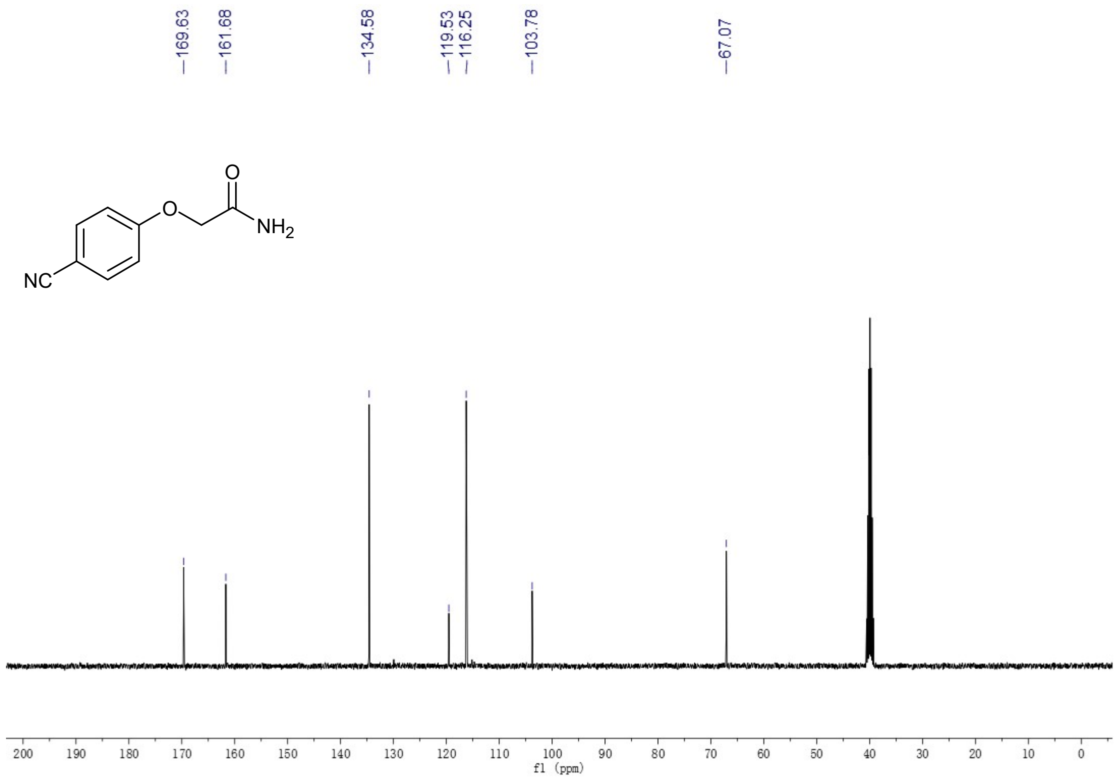

4.17 ^{1}H NMR and ^{13}C NMR of 3q

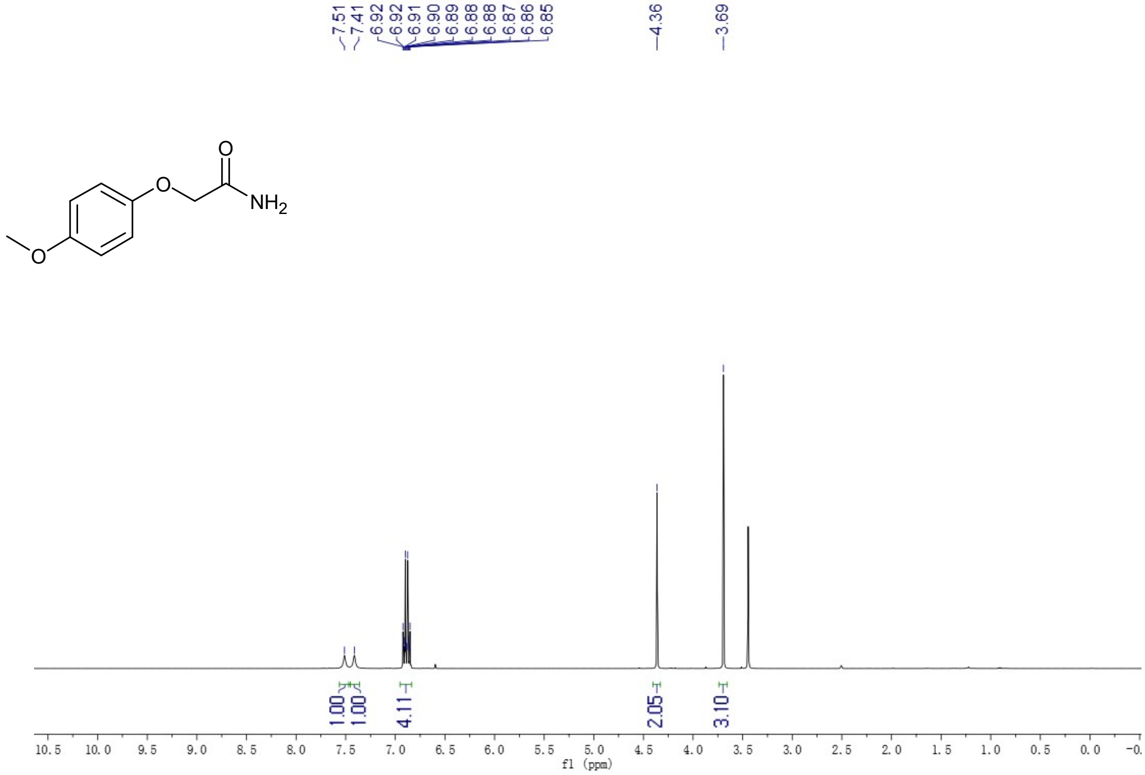

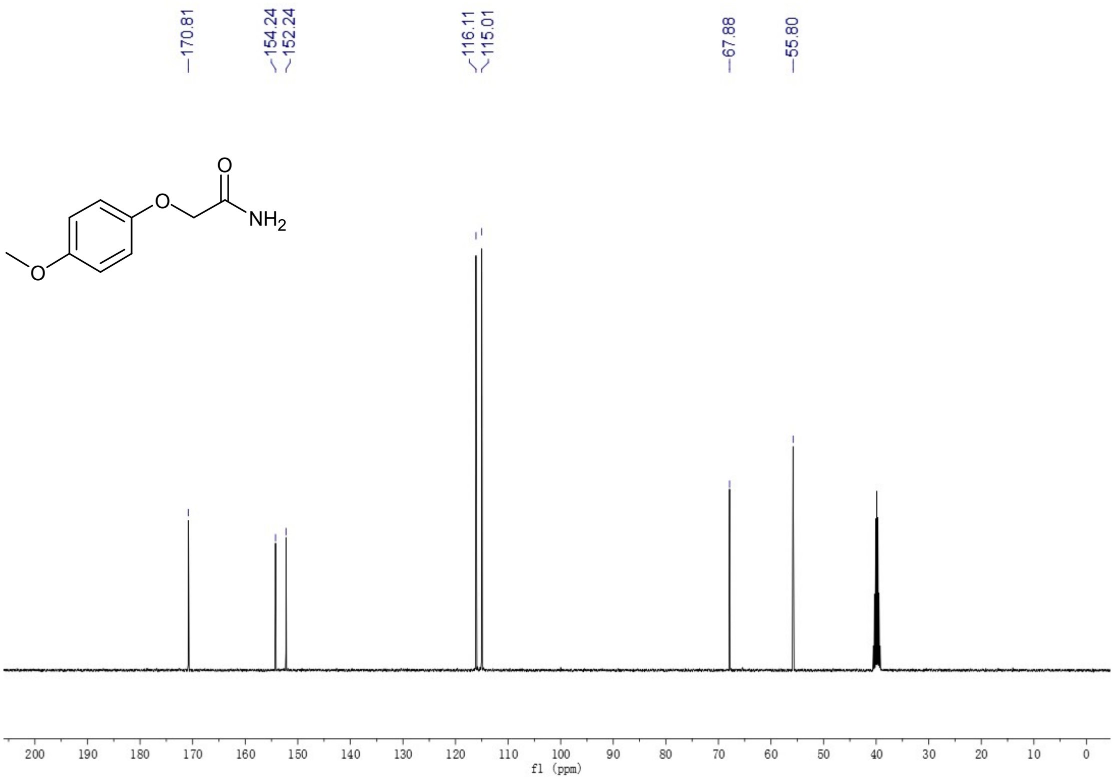

4.18 ^{1}H NMR and ^{13}C NMR of 3r

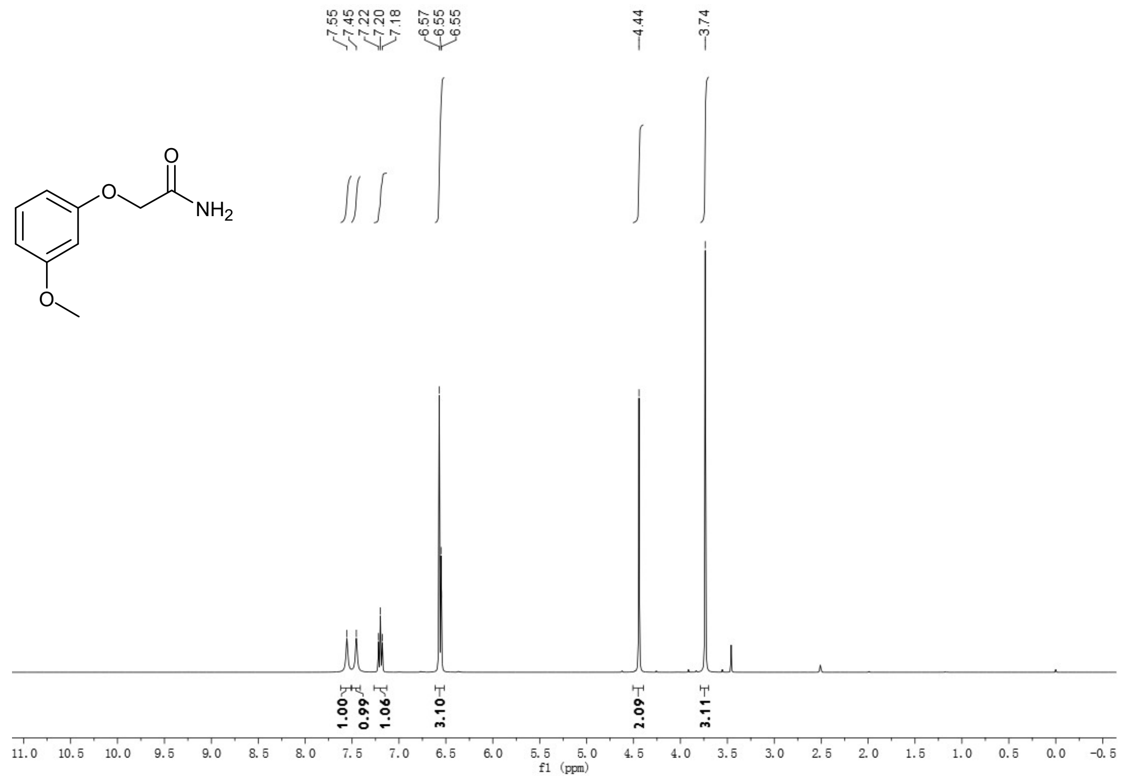

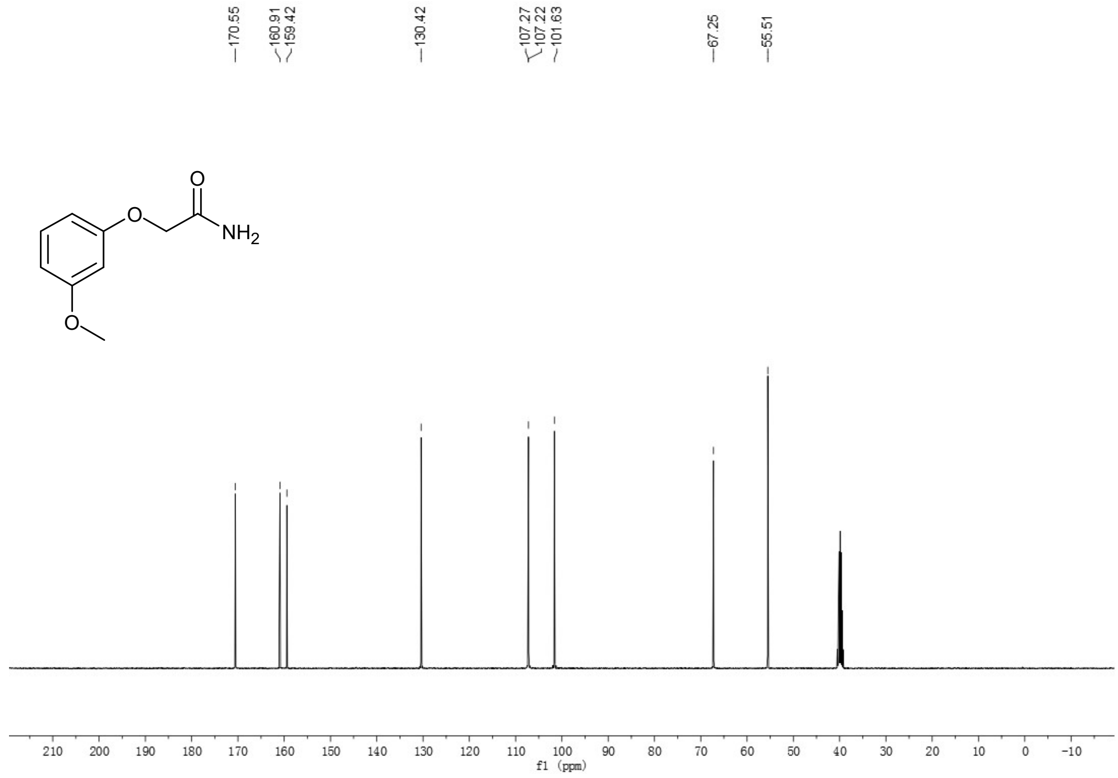

4.19 ^{1}H NMR and ^{13}C NMR of 3s

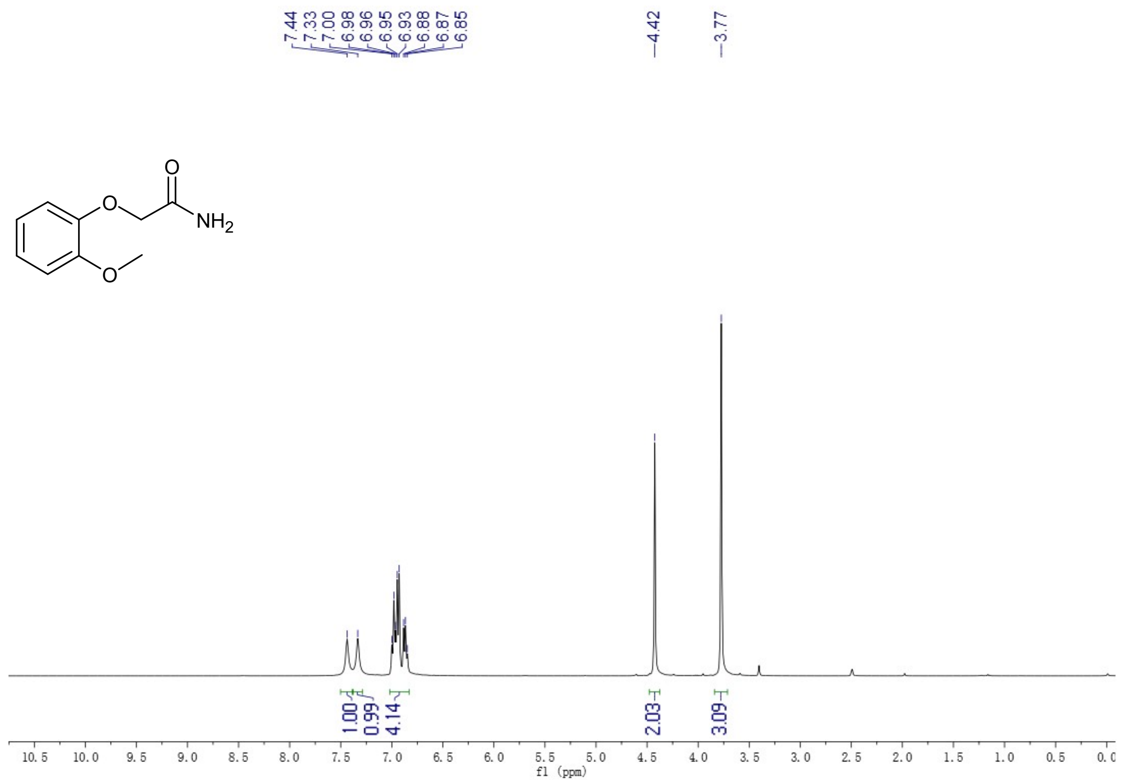
7.44
7.33
7.00
6.98
6.96
6.95
6.93
6.88
6.87
6.85
4.42
3.77
NH2
1.00
0.99
4.14
2.03
3.09
f1 (ppm)

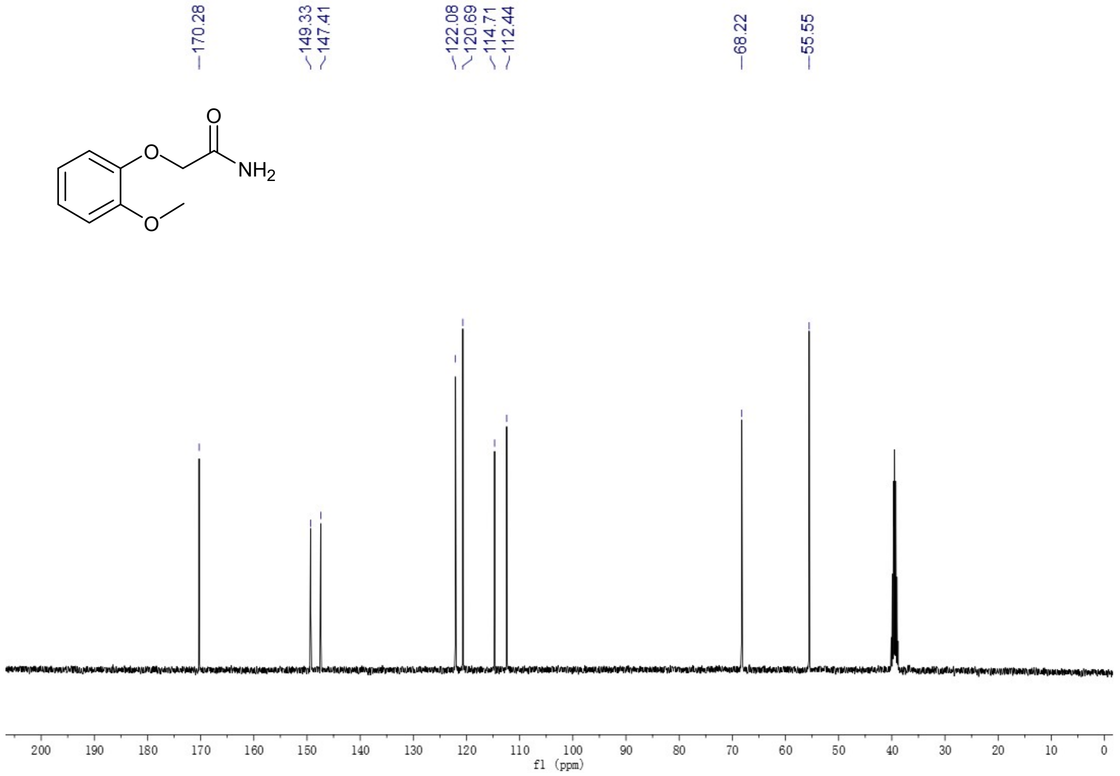
170.28
149.33
147.41
122.08
120.69
114.71
112.44
68.22
55.55
NH2
f1 (ppm)

4.20 ^{1}H NMR and ^{13}C NMR of 3t

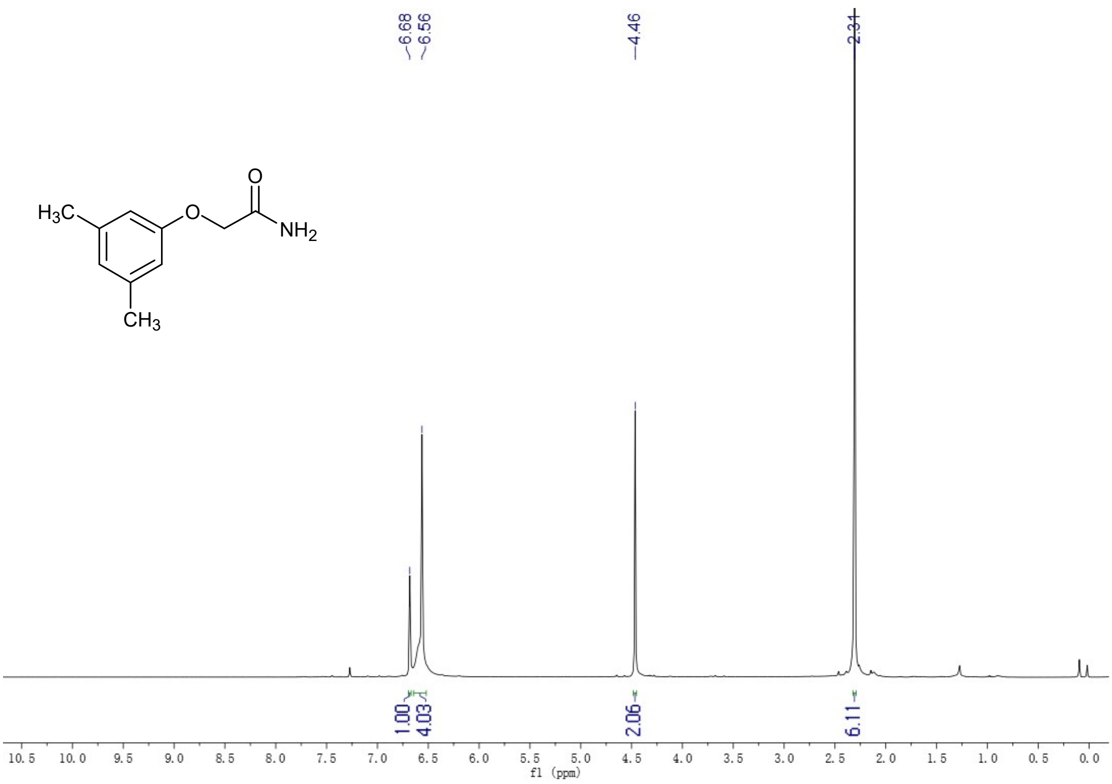

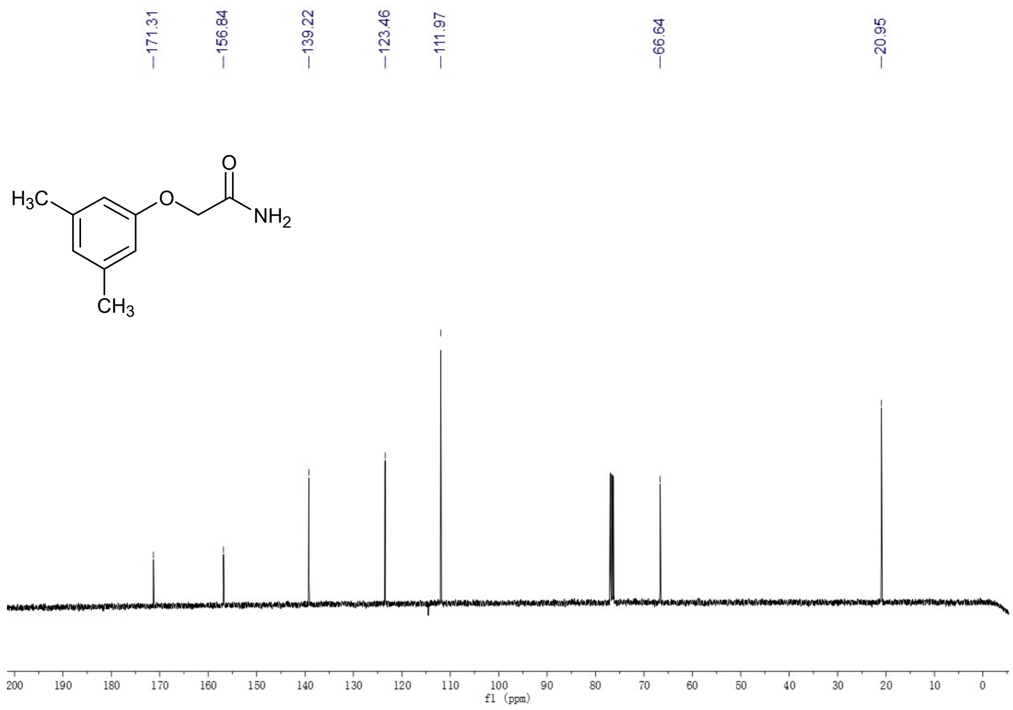

4.21 ^{1}H NMR and ^{13}C NMR of 3u

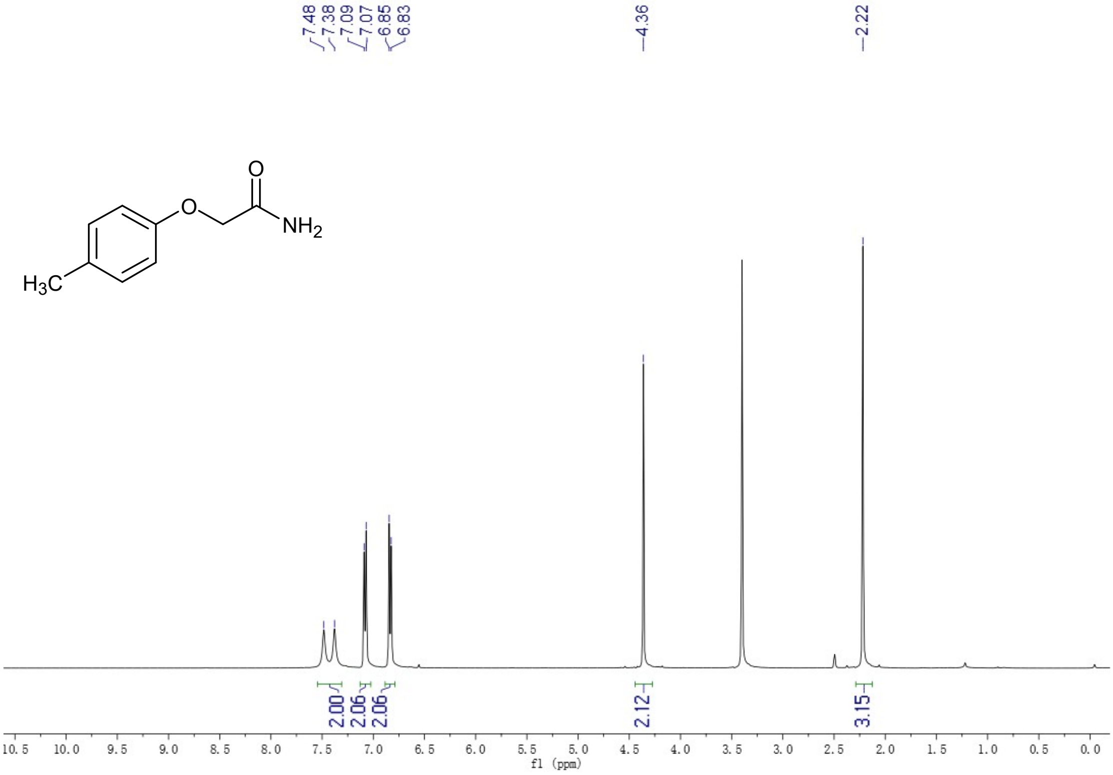

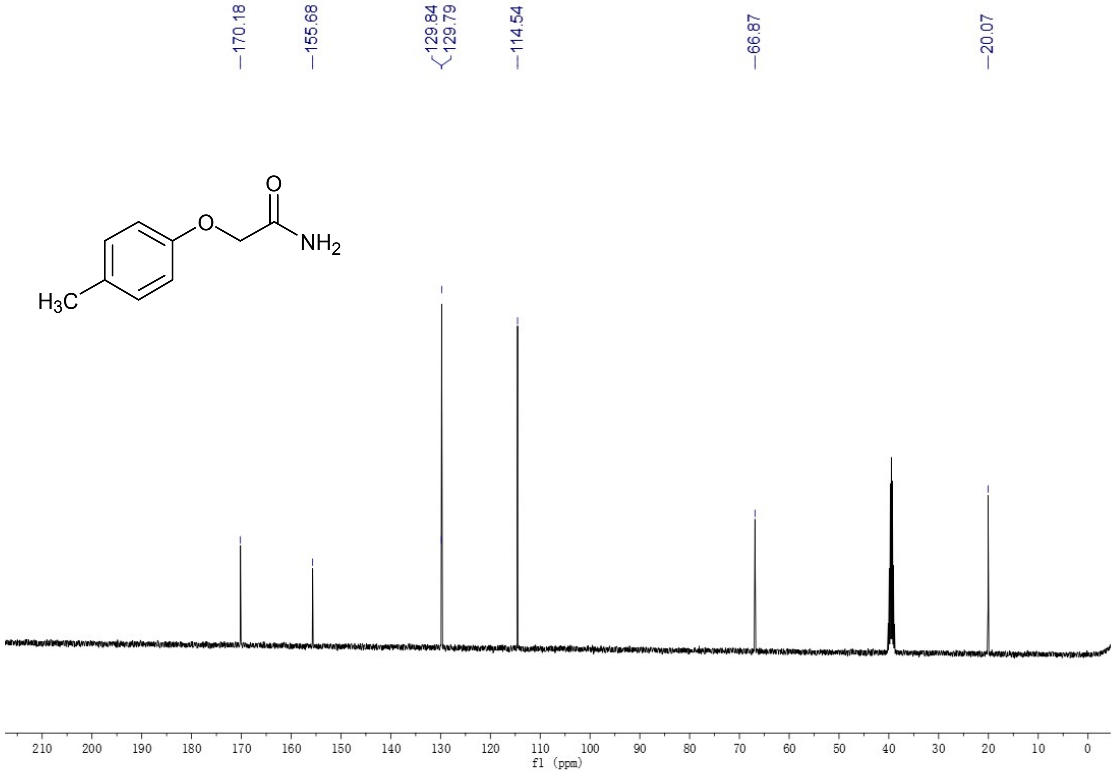

4.22 ^{1}H NMR and ^{13}C NMR of 3v

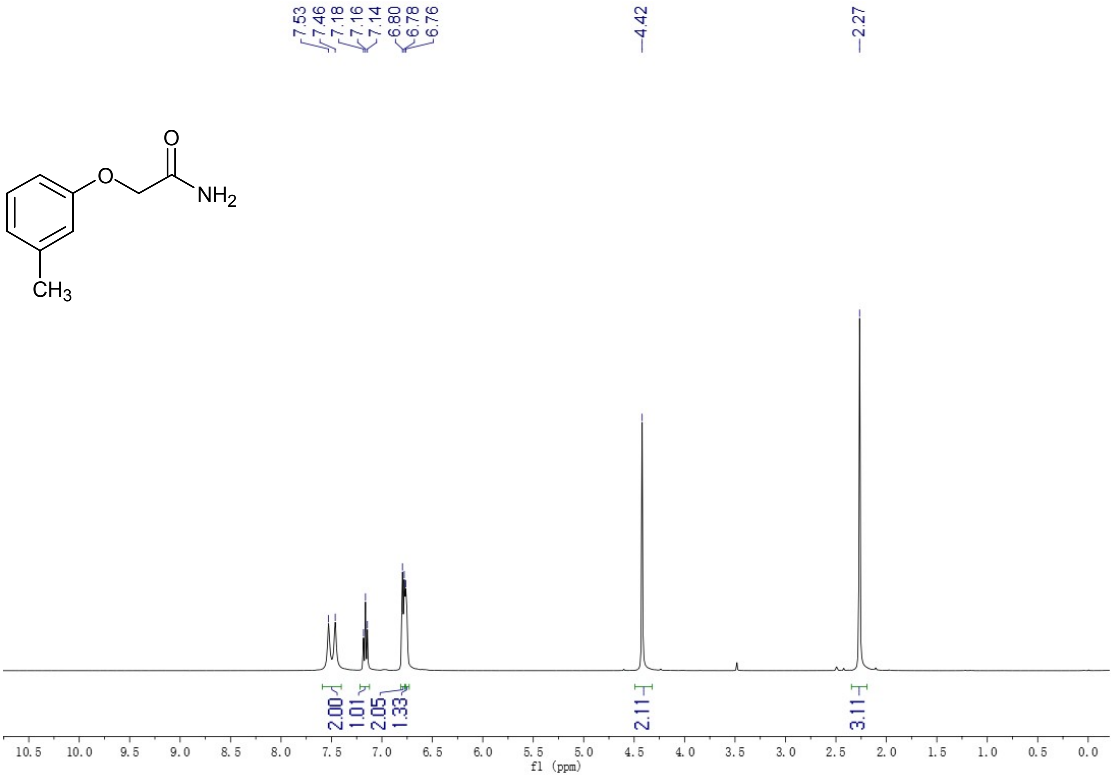

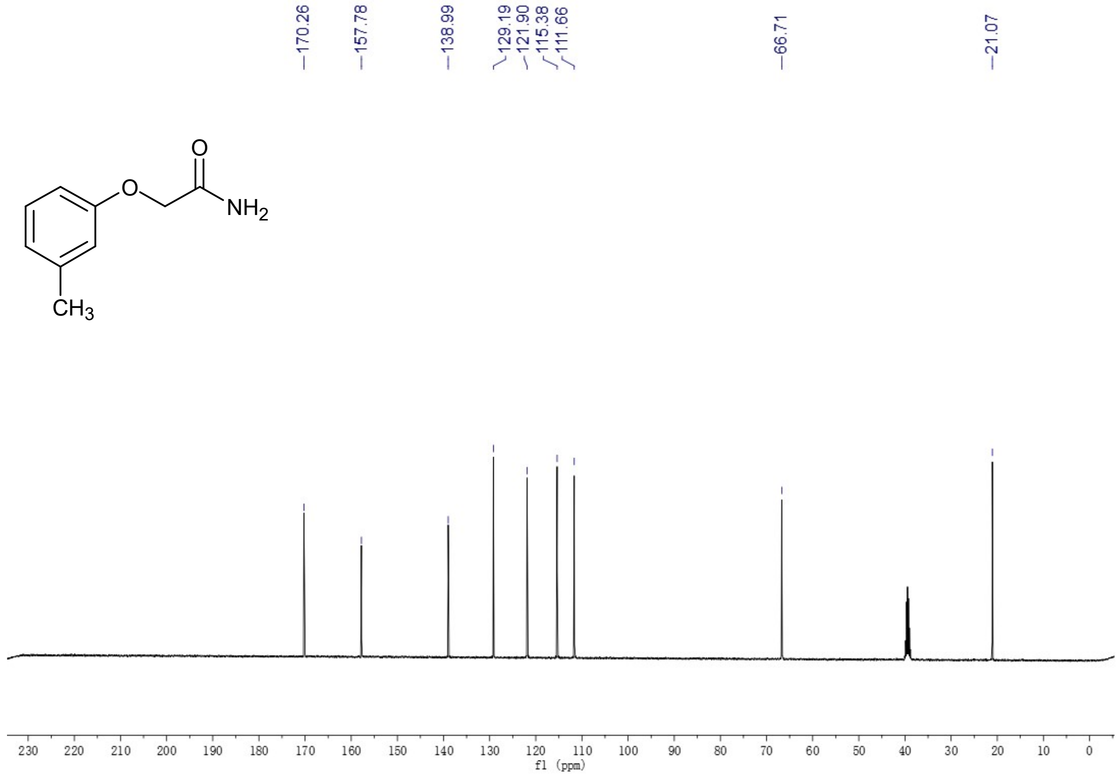

4.23 ^{1}H NMR and ^{13}C NMR of 3w

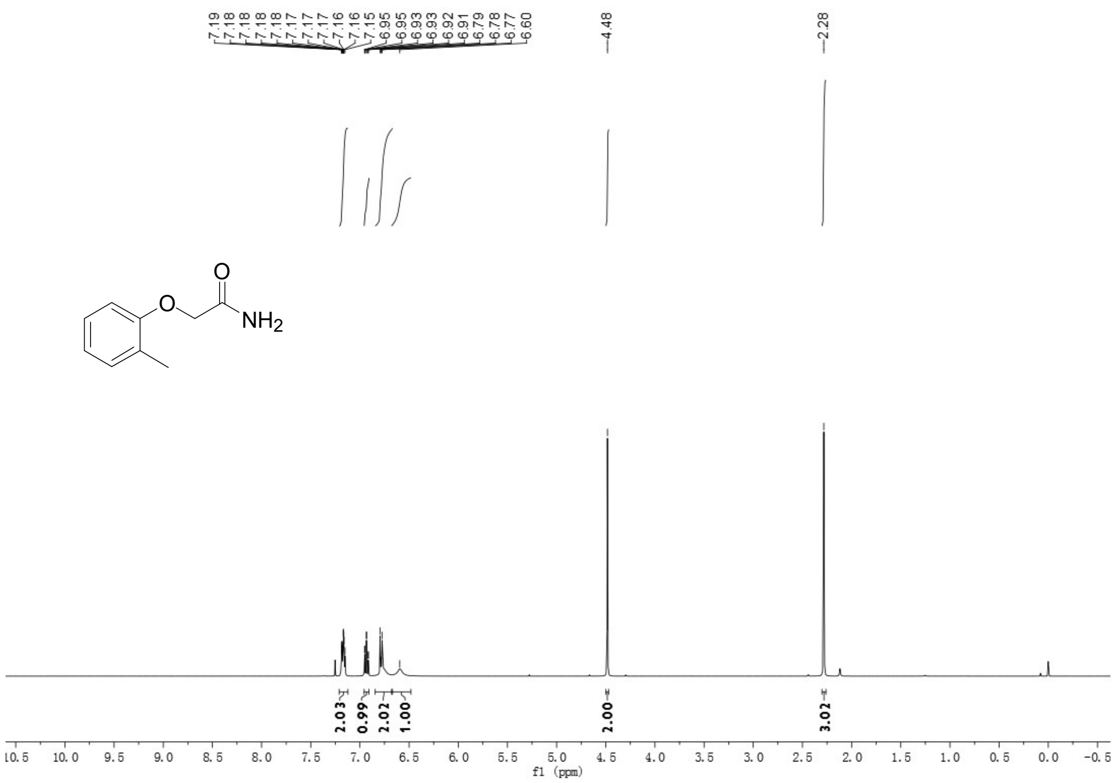

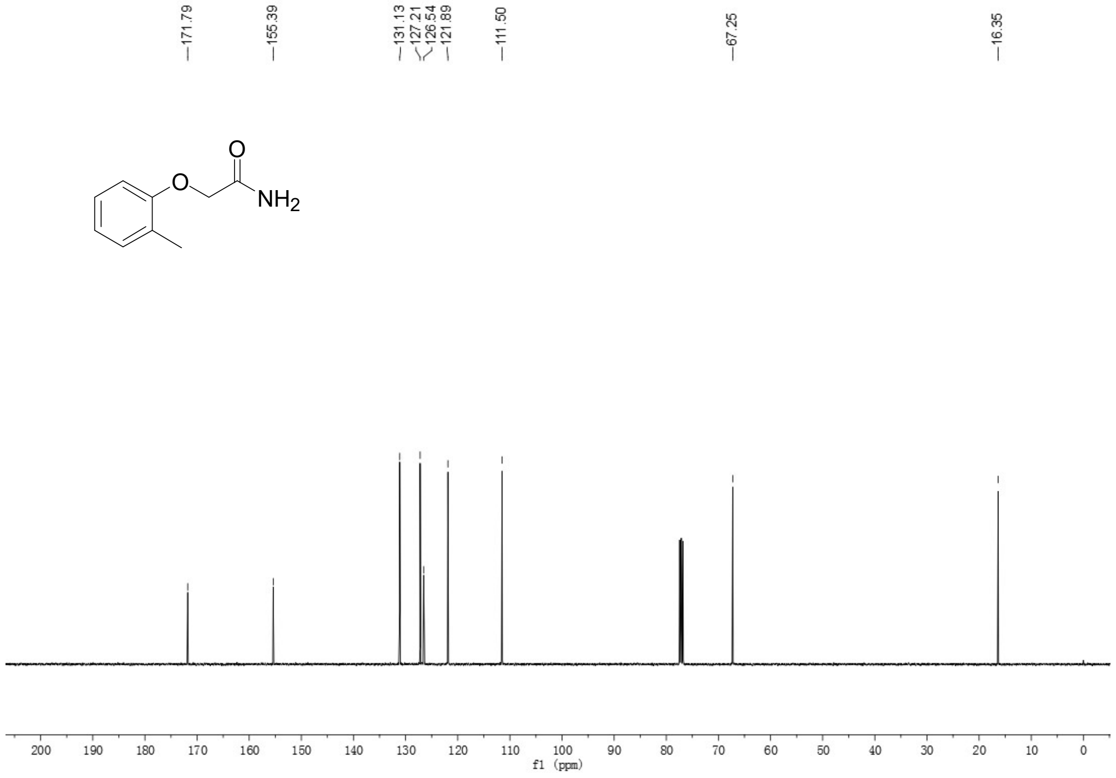

4.24 ^{1}H NMR and ^{13}C NMR of 3x

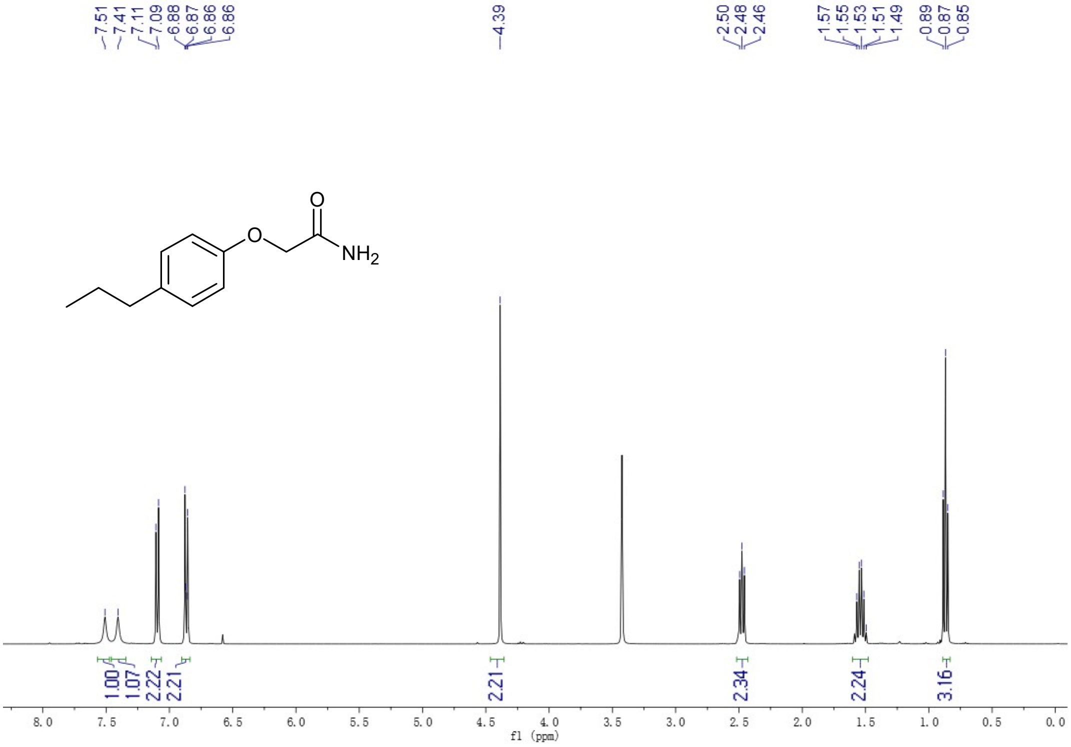

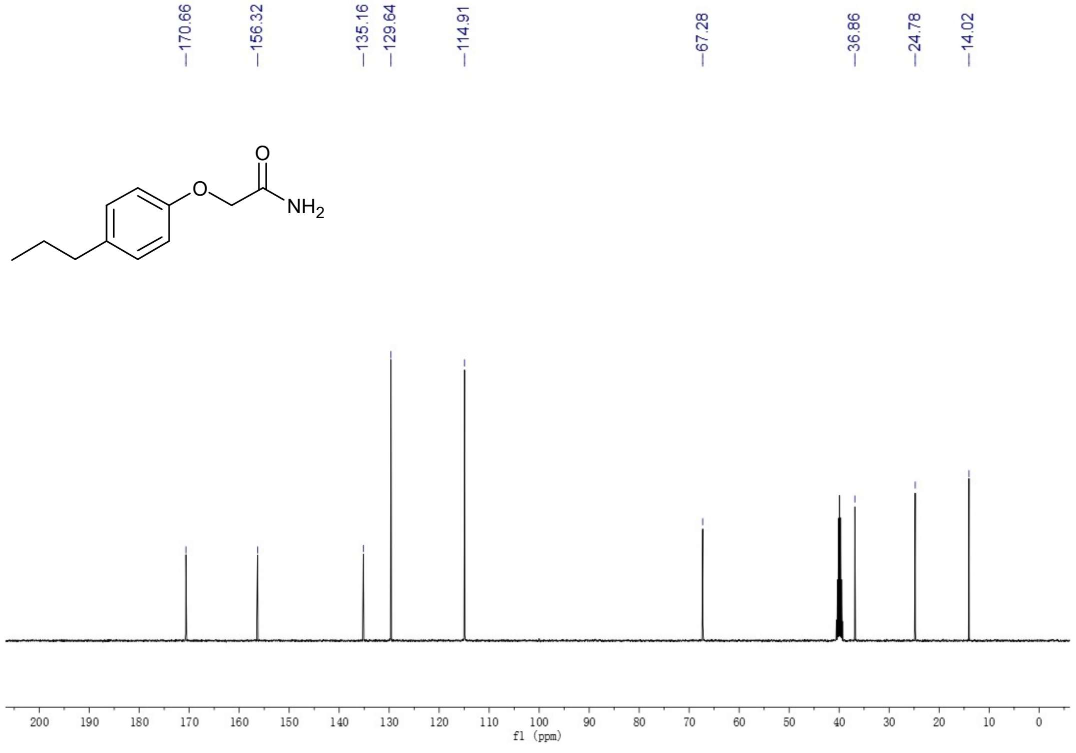

4.25 ^{1}H NMR and ^{13}C NMR of 3y

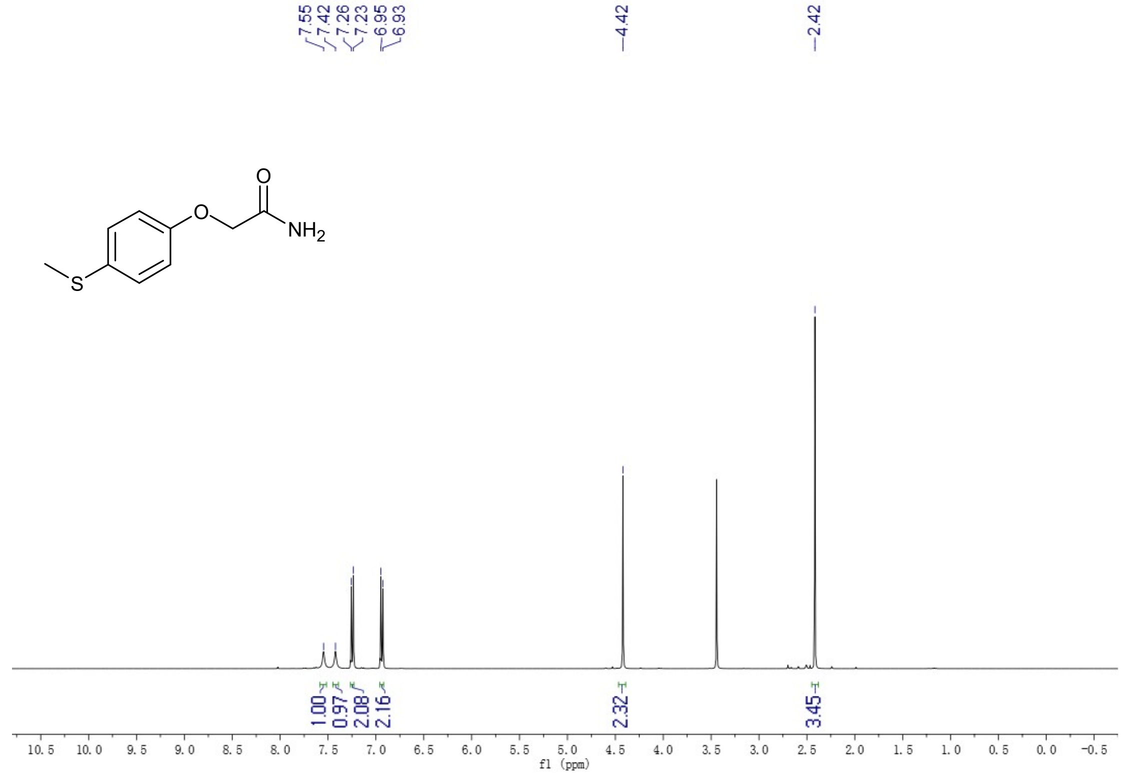
7.55
7.42
7.26
7.23
6.95
6.93
4.42
2.42
O
NH2
S
1.00
0.97
2.08
2.16
2.32
3.45
10.5 10.0 9.5 9.0 8.5 8.0 7.5 7.0 6.5 6.0 5.5 5.0 4.5 4.0 3.5 3.0 2.5 2.0 1.5 1.0 0.5 0.0 -0.5
f1 (ppm)

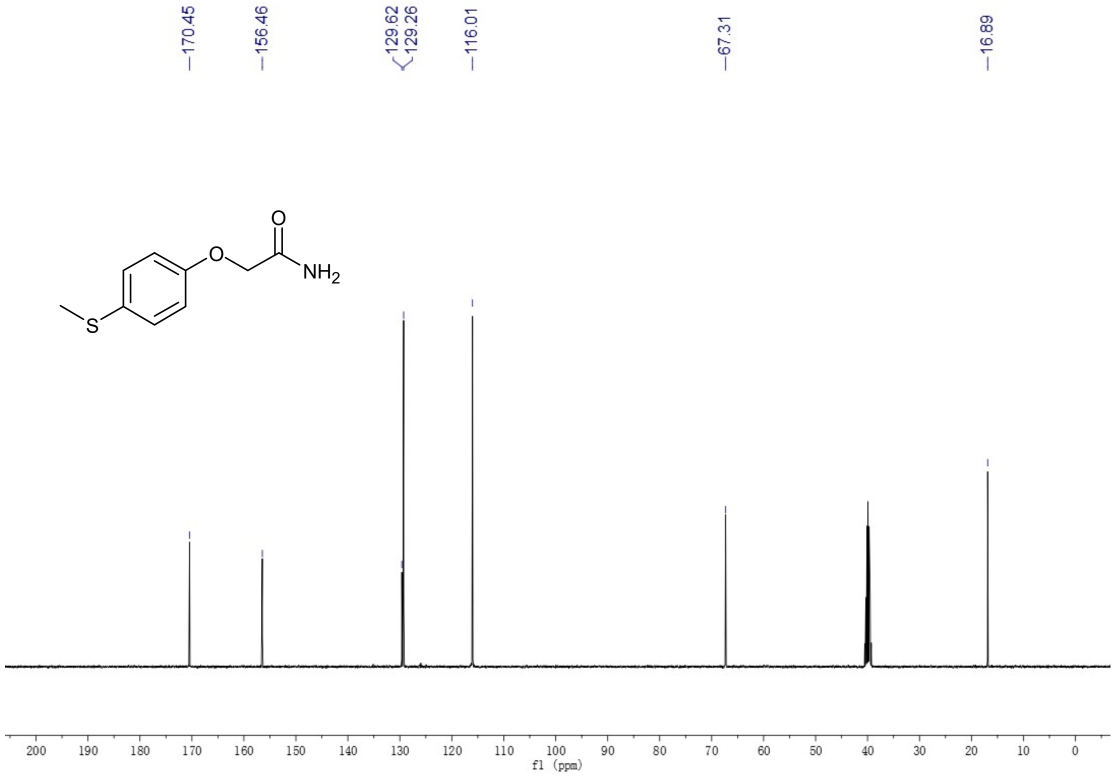
170.45
156.46
129.62
129.26
116.01
67.31
16.89
O
NH2
S
200 190 180 170 160 150 140 130 120 110 100 90 80 70 60 50 40 30 20 10 0
f1 (ppm)
